# Association of Body Shape Index (ABSI) with cardio-metabolic risk factors: A cross-sectional study of 6081 Caucasian adults

**DOI:** 10.1371/journal.pone.0185013

**Published:** 2017-09-25

**Authors:** Simona Bertoli, Alessandro Leone, Nir Y. Krakauer, Giorgio Bedogni, Angelo Vanzulli, Valentino Ippocrates Redaelli, Ramona De Amicis, Laila Vignati, Jesse C. Krakauer, Alberto Battezzati

**Affiliations:** 1 International Center for the Assessment of Nutritional Status (ICANS), Department of Food, Environmental and Nutritional Sciences (DeFENS), University of Milan, Milan, Italy; 2 Department of Civil Engineering, The City College of New York, New York, NY, United States of America; 3 Niguarda Cancer Center, Niguarda Cà Granda Hospital, Milan, Italy; 4 Department of Oncology and Hemato-oncology, University of Milan, Milan Italy; 5 Metro Detroit Diabetes and Endocrinology, Southfield, MI, United States of America; Johns Hopkins University Bloomberg School of Public Health, UNITED STATES

## Abstract

A Body Shape Index (ABSI) was specifically developed as a transformation of waist circumference (WC), statistically independent of BMI to better evaluate the relative contribution of WC to central obesity and clinical outcomes. Previous studies have found ABSI is associated with total mortality and cardiovascular events. However, no study has specifically evaluated the joint contribution of ABSI and BMI to cardio-metabolic outcomes (high triglycerides, low HDL, high fasting glucose and high blood pressure). With this aim, we performed a retrospective study on 6081 Caucasian adults. Subjects underwent a medical interview, anthropometric measurements, blood sampling, measurement of blood pressure, and measurement of visceral abdominal fat thickness (VAT) by ultrasound. Generalized linear models (GLM) were used to evaluate the sex and age adjusted association of ABSI with binary and continuous cardio-metabolic risk factors. Four pre-specified GLM were evaluated for each outcome: M1 = ABSI, BMI and ABSI*BMI interaction, M2 = ABSI and BMI, M3 = ABSI alone and M4 = BMI alone. Bayesian Information Criterion (BIC) was calculated and used to identify the best predictive model. ABSI and BMI contributed independently to all outcomes. Compared to BMI alone, the joint use of BMI and ABSI yielded significantly improved associations for having high triglycerides (BIC = 5261 vs. 5286), low HDL (BIC = 5371 vs. 5381), high fasting glucose (BIC = 6328 vs. 6337) but not high blood pressure (BIC = 6580 vs. 6580). The joint use of BMI and ABSI was also more strongly associated with VAT than BMI alone (BIC = 22930 vs. 23479). In conclusion, ABSI is a useful index for evaluating the independent contribution of WC, in addition to that of BMI, as a surrogate for central obesity on cardio-metabolic risk.

## Introduction

A Body Shape Index (ABSI) is calculated by dividing waist circumference (WC) by its estimate obtained from allometric regression of weight and height [1]. ABSI was designed to be minimally associated with weight, height and body mass index (BMI) so that it can be used together with BMI to disentangle the independent contribution of WC and BMI to cardio-metabolic outcomes [1–3].

ABSI is a predictor of total mortality, as reported by several cohort observational studies with follow-ups ranging from 5 to 25 years [1, 4–7]. ABSI also predicts incident cardiovascular disease (CVD) [8] with an accuracy similar to that of common laboratory measurements [9]. The fact that ABSI predicts CVD and mortality suggests that it has some potential for being incorporated into clinical guidelines in place of WC and together with BMI [10, 11]. The advantage of ABSI over WC is that, by design, it allows to establish the separate contribution of BMI and the so adjusted WC to morbidity and mortality.

There is, however, limited research on the association of ABSI with established cardio-metabolic disease (CMD) risk factors. In one study, ABSI was positively associated with serum insulin and C-reactive protein in men [12]. Some studies have found that ABSI was less strongly associated than BMI with established CVD risk factors [13–15]. However, very few studies have evaluated the joint contribution of BMI and ABSI to CMD risk factors. This is important because the very reason why ABSI was developed was to allow researchers to determine the separate contribution of BMI and the so adjusted WC to disease.

Studies of body composition have shown that ABSI is positively associated with fat mass and negatively associated with fat-free mass [16]. Also, in patients with type 2 diabetes mellitus, ABSI is positively associated with visceral fat [17]. The metabolic syndrome (MS) is positively associated with visceral fat [18–21] and negatively associated with muscle mass and gluteo-femoral fat [22, 23]. There are presently no data on the association of ABSI with MS and its components, i.e. high triglycerides, low HDL cholesterol, high blood pressure and high fasting glucose.

We performed a retrospective study of a large sample of Caucasian adults to quantify the separate and joint contribution of ABSI to MS components [18] and visceral abdominal tissue thickness (VAT), independently of sex, age and BMI.

## Materials and methods

### Study design

6081 consecutive Caucasian subjects (4384 women, 72%) were retrospectively studied at International Center for the Assessment of Nutritional Status (ICANS), University of Milan, between September 2010 and June 2015. All subjects were enrolled because of their interest to undergo a structured nutritional assessment. Criteria for inclusion into the study were: 1) age ≥ 18 years; 2) body mass index (BMI) ≥ 18.5 kg·m^-2^. Exclusion criteria were: having acute infective, neurological, gastrointestinal, cardiac, renal and pulmonary disorders [24], use of medications known to cause lipodystrophy such as steroids, other immunosuppressive and anti-retroviral agents, and presence of scars in the measurement area of VAT [25]. On the same morning, the fasting subjects underwent a medical interview, an anthropometric assessment, a measurement of blood pressure, an abdominal ultrasonography (US), and blood sampling. The study was performed in accordance with the Declaration of Helsinki and the subjects gave their written informed consent. The study procedures were approved by the Ethical Committee of Milan University.

### Anthropometric and clinical assessment

Weight and height were measured following international guidelines [26]. BMI was calculated and obesity was classified following the NIH guidelines [27]. WC was measured at the midpoint between the last rib and the iliac crest. ABSI (m^11/6^ kg^-2/3^) and its standard deviation score (SDS) were calculated using the following formula:

$$ABSI=\frac{WC}{{BMI}^{2/3}\;{Height}^{1/2}}.$$ (1)

Resting blood pressure was measured twice in sitting position after participants had rested for at least five minutes following the JNC-7 guidelines [28].

### Abdominal ultrasonography

Abdominal US was performed by the same operator using a Logiq 3 Pro instrument equipped with a 3.5 MHz convex-array probe (GE Healthcare, Milwaukee, WI, USA). VAT, defined as the distance between the anterior wall of the aorta and the posterior surface of the rectus abdominis muscle, was measured 1 cm above the umbilicus at end-expiration [29]. The VAT measurement was performed three times and the mean of the three measures was used for analysis. The within-day intra-operator coefficient of variation (CV) for repeated measures of VAT at ICANS is 0.8% [25, 30, 31].

### Laboratory assessment

Fasting blood samples were drawn between 8:30 and 9:00 AM and analyzed in the same morning at the ICANS internal laboratory. Glucose, triglycerides, total cholesterol, HDL-cholesterol and LDL-cholesterol were measured by means of an enzymatic method (Cobas Integra 400 Plus, Roche Diagnostics, Rotkreuz, Switzerland) with intra-and inter-assay CV < 2% [32].

### Metabolic syndrome

MS was diagnosed using the harmonized international definition [18]. In detail, high WC was defined as WC ≥ 102 cm in men and ≥ 88 cm in women; low HDL as HDL < 1.04 mmol·L^-1^ in men and < 1.29 mmol·L^-1^ in women; high triglycerides as triglycerides ≥ 1.7 mmol·L^-1^ or treatment with triglycerides-lowering drugs, high blood pressure as systolic blood pressure ≥ 130 mmHg or diastolic blood pressure ≤ 85 mmHg or treatment with pressure lowering drugs, and high glucose as glucose ≥ 5.6 mmol·L^-1^ or treatment with glucose-lowering drugs, and MS as ≥ 3 of the above.

### Statistical analysis

Most continuous variables were not Gaussian-distributed and all are reported as 50^th^, 25^th^ and 75^th^ percentiles. Categorical variables are reported as numbers and proportions. We evaluated the association of ABSI with the binary outcomes (high triglycerides, low HDL, high blood pressure and high fasting glucose; 0 = no, 1 = yes) and the continuous outcome (VAT, cm) using generalized linear models (GLM). The GLM had a binomial family and a logit link for the binary outcomes and a Gaussian family and an identity link for the continuous outcome [33]. Four pre-specified GLM were evaluated for each outcome with the following combinations of predictors:

1. ABSI (continuous, SDS), BMI (discrete, 0 = normal weight; 1 = overweight; 2 = class 1 obesity; 3 = class 2 obesity; 4 = class 3 obesity), ABSI·BMI interaction (continuous X discrete), sex (discrete; 0 = female; 1 = male), age (discrete, 0 = 19 to 29 years; 1 = 30 to 39 years; 2 = 40 to 49 years; 3 = 50 to 59 years; 4 = 60 to 69 years; 5 = 70 to 76 years).
2. same as #1 without the ABSI·BMI interaction.
3. same as #2 without BMI.
4. same as #2 without ABSI.

Model #1 tests the joint contribution of ABSI and BMI if ABSI and BMI do interact, taking into account the effect of sex and age. Model #2 tests the joint contribution of ABSI and BMI if no interaction is present, taking into account the effect of sex and age. Model #3 evaluates the effect of ABSI alone, taking into account the effect of sex and age. Lastly, Model #4 evaluates the effect of BMI alone, taking into account the effect of sex and age. Standard diagnostics of model fit for GLM were used and the Bayesian information criterion (BIC) was calculated [33, 34] and used to perform a relative comparison of the models. “Weak evidence” in favor of the model with the lower BIC is said to exist when the BIC difference (ΔBIC) is ≤2; “positive evidence” when 6 > ΔBIC > 2; “strong evidence” when 6 ≤ ΔBIC < 10; and “very strong evidence” when ΔBIC ≥ 10 [34]. Effects plots were obtained for each model at pre-specified values of ABSI SDS (5^th^, 25^th^, 50^th^, 75^th^ and 95^th^ internal percentiles) to allow a clinically meaningful interpretation of the results [33]. Statistical analysis was performed using Stata 14.2 (Stata Corporation, College Station, TX, USA).

## Results

**Table 1**gives the anthropometric and laboratory measurements of the subjects. The median BMI was 28.0 kg·m^-2^ in women and 30.3 kg·m^-2^ in men. The median SDS of ABSI in this population corresponds to the 40^th^ NHANES 1999–2004 percentile in women and to the 53^th^ NHANES percentile in men [1, 2].

**Table 1:** Measurements of the study subjects.

	Women (*n = 4384*)			Men (*n = 1697*)			Total (*n = 6081*)		
	P50	P25	P75	P50	P25	P75	P50	P25	P75
Age (years)	46	38	56	47	39	56	47	38	56
Weight (kg)	72.8	64.9	82.6	93	84.2	104	78.1	67.7	90.5
Height (m)	1.61	1.57	1.66	1.75	1.71	1.79	1.64	1.59	1.71
BMI (kg m^-2^)	28	24.9	31.7	30.3	27.7	33.3	28.7	25.6	32.3
BMI (SDS NHANES)	-0.09	-0.52	0.48	0.38	-0.12	0.98	0.04	-0.43	0.64
ABSI (m^11/6^ kg^-2/3^)	0.08	0.08	0.08	0.08	0.08	0.09	0.08	0.08	0.08
ABSI (SDS NHANES)	-0.25	-0.81	0.27	0.08	-0.46	0.64	-0.16	-0.73	0.38
Waist circumference (cm)	92	84	101	106	99.1	115	96	86.8	106
VAT (cm)	4.2	3	5.8	7.5	5.6	9.3	4.9	3.4	7.2
Glucose (mmol l^-1^)	5.11	4.77	5.44	5.44	5.11	5.94	5.16	4.83	5.61
Triglycerides (mmol l^-1^)	0.95	0.71	1.3	1.34	0.96	1.91	1.04	0.77	1.47
Cholesterol (mmol l^-1^)	5.46	4.82	6.15	5.43	4.76	6.15	5.46	4.81	6.15
LDL (mmol l^-1^)	3.34	2.77	3.96	3.57	2.95	4.16	3.39	2.82	4.03
HDL (mmol l^-1^)	1.63	1.4	1.89	1.22	1.03	1.42	1.5	1.27	1.78
Systolic blood pressure (mm Hg)	120	110	130	130	120	140	120	110	130
Diastolic blood pressure (mm Hg)	80	70	80	80	80	90	80	70	85

Abbreviations: P50 = 50^th^ percentile (median); P25 = 25^th^ percentile; P75 = 75^th^ percentile; BMI = body mass index; ABSI = a body shape index; SDS = standard deviations scores; VAT = visceral adipose tissue; LDL = low-density lipoprotein; HDL = high-density lipoprotein. Conversion factors: Triglycerides: mg/dL = 89 * mmol/L; Total, HDL and LDL cholesterol: mg/dL = 38.67 * mmol/L; Glucose: mg/dL = 18 * mmol/L

**Table 2**gives the distribution of age, body mass index and MS and its components. 20.3% of the subjects had normal weight, 39.7% were overweight, 27.4% had class 1 obesity, 9.9% had class 2 obesity, and 2.6% had class 3 obesity. High waist circumference was detected in 63.6% of all subjects, high triglycerides in 17.4%, low HDL in 17.0%, high blood pressure in 44.3% and high fasting glucose in 28.7%. MS was present in 27.0% of the subjects.

**Table 2:** Distribution of age, body mass index and the metabolic syndrome and its components in the study subjects.

	Women		Men		Total	
	N	%	N	%	N	%
**Age**						
19 to 29 years	428	9.8	101	6	529	8.7
30 to 39 years	859	19.6	330	19.4	1189	19.6
40 to 49 years	1313	29.9	533	31.4	1846	30.4
50 to 59 years	1042	23.8	410	24.2	1452	23.9
60 to 69 years	585	13.3	256	15.1	841	13.8
70 to 76 years	157	3.6	67	3.9	224	3.7
**BMI NIH classification**						
Normal weight	1108	25.3	126	7.4	1234	20.3
Overweight	1748	39.9	669	39.4	2417	39.7
Class 1 obesity	1037	23.7	631	37.2	1668	27.4
Class 2 obesity	384	8.8	218	12.8	602	9.9
Class 3 obesity	107	2.4	53	3.1	160	2.6
**High waist circumference**						
No	1637	37.3	574	33.8	2211	36.4
Yes	2747	62.7	1123	66.2	3870	63.6
**High triglycerides**						
No	3876	88.4	1145	67.5	5021	82.6
Yes	508	11.6	552	32.5	1060	17.4
**Triglyceride lowering drugs**						
No	4359	99.4	1673	98.6	6032	99.2
Yes	25	0.6	24	1.4	49	0.8
**High triglycerides OR triglyceride-lowering drugs**						
No	3857	88	1130	66.6	4987	82
Yes	527	12	567	33.4	1094	18
**Low HDL**						
No	3715	84.7	1330	78.4	5045	83
Yes	669	15.3	367	21.6	1036	17
**High blood pressure**						
No	2822	64.4	567	33.4	3389	55.7
Yes	1562	35.6	1130	66.6	2692	44.3
**Blood pressure lowering drugs**						
No	3726	85	1230	72.5	4956	81.5
Yes	658	15	467	27.5	1125	18.5
**High blood pressure OR blood pressure-lowering drugs**						
No	2607	59.5	468	27.6	3075	50.6
Yes	1777	40.5	1229	72.4	3006	49.4
**High fasting glucose**						
No	3409	77.8	929	54.7	4338	71.3
Yes	975	22.2	768	45.3	1743	28.7
**Glucose lowering drugs**						
No	4355	99.3	1654	97.5	6009	98.8
Yes	29	0.7	43	2.5	72	1.2
**High blood glucose OR glucose-lowering drugs**						
No	3404	77.6	927	54.6	4331	71.2
Yes	980	22.4	770	45.4	1750	28.8
**Metabolic syndrome score (including use of drugs)**						
0	1046	23.9	146	8.6	1192	19.6
1	1257	28.7	293	17.3	1550	25.5
2	1137	25.9	439	25.9	1576	25.9
3	674	15.4	474	27.9	1148	18.9
4	203	4.6	262	15.4	465	7.6
5	67	1.5	83	4.9	150	2.5
**Metabolic syndrome (including use of drugs)**						
No	3440	78.5	878	51.7	4318	71
Yes	944	21.5	819	48.3	1763	29
**Statins**						
No	4221	96.3	1574	92.8	5795	95.3
Yes	163	3.7	123	7.2	286	4.7

**Tables 3–6**give the regression coefficients and BIC for the 4 GLM used to investigate the association between the outcomes of interest (high triglycerides, low HDL, high blood pressure and high fasting glucose) and ABSI. The full models (models A1 to D1) allowed to explore the existing interaction between ABSI and BMI in the prediction of the outcomes of interest. No ABSI·BMI interaction was detected for high triglycerides (model A1) and blood pressure (model C1), whereas such interaction was present for low HDL (model B1) and, to a lesser extent, for high glucose (model D1). ABSI and BMI contributed independently to all outcomes, even when the interaction was not accounted for (models A2 to D2). ABSI alone, when BMI was not included in the models (models A3 to D3) provided the weakest prediction of all outcomes of interest. Compared to BMI alone (models A4 to D4), the joint use of BMI and ABSI was more strongly associated with the probability of having high triglycerides (BIC = 5261 for model A2 vs. 5286 for model A4), low HDL (BIC = 5371 for model B2 vs. 5381 for model B4), high fasting glucose (BIC = 6328 for model D2 vs. 6337 for model D4) but not high blood pressure (BIC = 6580 for model C2 vs. 6580 for model C4).

**Table 3:** Generalized linear models developed for studying the association between ABSI, BMI and high triglycerides.

	High triglycerides OR triglyceride-lowering drugs			
	A1	A2	A3	A4
**ABSI**				
ABSI (SDS NHANES)	0.36*	0.26***	0.28***	
	[0.07,0.65]	[0.17,0.34]	[0.19,0.36]	
**BMI classes**				
Normal weight	Ref.	Ref.		Ref.
Overweight	0.68***	0.70***		0.73***
	[0.42,0.95]	[0.44,0.96]		[0.47,0.99]
Obesity 1	1.19***	1.20***		1.24***
	[0.92,1.45]	[0.94,1.46]		[0.97,1.50]
Obesity 2 and 3	1.35***	1.38***		1.39***
	[1.06,1.64]	[1.09,1.67]		[1.11,1.68]
**BMI-ABSI interaction**				
Normal # ABSI (SDS NHANES)	Ref.			
Overweight # ABSI (SDS NHANES)	0.00			
	[-0.32,0.33]			
Obesity 1 # ABSI (SDS NHANES)	-0.13			
	[-0.46,0.19]			
Obesity 2 and 3 # ABSI (SDS NHANES)	-0.30			
	[-0.65,0.06]			
**Age classes**				
19 to 29 years	Ref.	Ref.	Ref.	Ref.
30 to 39 years	-0.08	-0.07	-0.08	-0.05
	[-0.39,0.24]	[-0.39,0.25]	[-0.39,0.23]	[-0.36,0.27]
40 to 49 years	0.11	0.12	0.15	0.15
	[-0.19,0.40]	[-0.18,0.41]	[-0.14,0.44]	[-0.14,0.44]
50 to 59 years	0.24	0.24	0.32*	0.28
	[-0.06,0.53]	[-0.06,0.54]	[0.03,0.62]	[-0.02,0.57]
60 to 69 years	0.40*	0.42**	0.56***	0.43**
	[0.08,0.71]	[0.10,0.73]	[0.25,0.87]	[0.12,0.74]
70 to 76 years	0.47*	0.49*	0.66**	0.49*
	[0.06,0.89]	[0.07,0.90]	[0.24,1.07]	[0.08,0.90]
**Sex**				
Women	Ref.	Ref.	Ref.	Ref.
Men	1.06***	1.05***	1.21***	1.13***
	[0.92,1.21]	[0.91,1.20]	[1.07,1.35]	[0.99,1.27]
Constant	-2.90***	-2.91***	-2.16***	-3.01***
	[-3.24,-2.55]	[-3.26,-2.57]	[-2.43,-1.89]	[-3.35,-2.68]
Observations	6081	6081	6081	6081
BIC	5281	5261	5370	5286

**Table 4:** Generalized linear models developed for studying the association between ABSI, BMI and low HDL.

	Low HDL			
	B1	B2	B3	B4
**ABSI**				
ABSI (SDS NHANES)	0.54***	0.19***	0.21***	
	[0.26,0.81]	[0.10,0.27]	[0.12,0.29]	
**BMI classes**				
Normal	Ref.	Ref.		Ref.
Overweight	0.73***	0.79***		0.81***
	[0.48,0.98]	[0.54,1.04]		[0.56,1.06]
Obesity 1	1.32***	1.38***		1.41***
	[1.06,1.57]	[1.12,1.63]		[1.15,1.66]
Obesity 2 and 3	1.58***	1.66***		1.67***
	[1.30,1.86]	[1.39,1.94]		[1.40,1.95]
**BMI-ABSI interaction**				
Normal # ABSI (SDS NHANES)	Ref.			
Overweight # ABSI (SDS NHANES)	-0.35*			
	[-0.66,-0.04]			
Obesity 1 # ABSI (SDS NHANES)	-0.35*			
	[-0.66,-0.04]			
Obesity 2 and 3 # ABSI (SDS NHANES)	-0.53**			
	[-0.86,-0.19]			
**Age classes**				
19 to 29 years	Ref.	Ref.	Ref.	Ref.
30 to 39 years	0.04	0.05	0.05	0.07
	[-0.24,0.31]	[-0.22,0.32]	[-0.21,0.32]	[-0.20,0.35]
40 to 49 years	-0.24	-0.22	-0.16	-0.18
	[-0.50,0.02]	[-0.48,0.04]	[-0.42,0.09]	[-0.44,0.08]
50 to 59 years	-0.44**	-0.42**	-0.30*	-0.38**
	[-0.71,-0.16]	[-0.70,-0.15]	[-0.56,-0.03]	[-0.65,-0.11]
60 to 69 years	-0.48**	-0.45**	-0.24	-0.43**
	[-0.78,-0.18]	[-0.75,-0.15]	[-0.53,0.05]	[-0.73,-0.13]
70 to 76 years	-0.48*	-0.46*	-0.22	-0.44*
	[-0.91,-0.05]	[-0.89,-0.03]	[-0.64,0.20]	[-0.87,-0.01]
**Sex**				
Women	Ref.	Ref.	Ref.	Ref.
Men	0.17*	0.16*	0.36***	0.22**
	[0.02,0.32]	[0.01,0.31]	[0.21,0.51]	[0.08,0.37]
Constant	-2.30***	-2.37***	-1.52***	-2.47***
	[-2.61,-2.00]	[-2.68,-2.07]	[-1.75,-1.29]	[-2.77,-2.17]
Observations	6081	6081	6081	6081
BIC	5388	5371	5552	5381

**Table 5:** Generalized linear models developed for studying the association between ABSI, BMI and high blood pressure.

	High blood pressure OR blood pressure-lowering drugs			
	C1	C2	C3	C4
**ABSI**				
ABSI (SDS NHANES)	0.18	0.11**	0.13***	
	[-0.00,0.37]	[0.04,0.19]	[0.06,0.20]	
**BMI classes**				
Normal weight	Ref.	Ref.		Ref.
Overweight	0.66***	0.68***		0.69***
	[0.48,0.84]	[0.51,0.86]		[0.52,0.87]
Obesity 1	1.43***	1.45***		1.46***
	[1.24,1.62]	[1.26,1.63]		[1.28,1.65]
Obesity 2 and 3	2.32***	2.34***		2.34***
	[2.07,2.57]	[2.10,2.57]		[2.10,2.58]
**BMI-ABSI interaction**				
Normal # ABSI (SDS NHANES)	Ref.			
Overweight # ABSI (SDS NHANES)	-0.12			
	[-0.34,0.10]			
Obesity 1 # ABSI (SDS NHANES)	-0.03			
	[-0.26,0.20]			
Obesity 2 and 3 # ABSI (SDS NHANES)	-0.07			
	[-0.36,0.22]			
**Age classes**				
19 to 29 years	Ref.	Ref.	Ref.	Ref.
30 to 39 years	0.34*	0.34*	0.31*	0.35*
	[0.07,0.60]	[0.07,0.60]	[0.06,0.56]	[0.08,0.61]
40 to 49 years	1.03***	1.03***	0.99***	1.05***
	[0.78,1.28]	[0.78,1.28]	[0.75,1.23]	[0.80,1.30]
50 to 59 years	1.70***	1.70***	1.68***	1.73***
	[1.45,1.96]	[1.45,1.96]	[1.44,1.93]	[1.47,1.98]
60 to 69 years	2.51***	2.51***	2.56***	2.53***
	[2.22,2.80]	[2.22,2.80]	[2.28,2.83]	[2.24,2.81]
70 to 76 years	3.15***	3.16***	3.23***	3.16***
	[2.69,3.62]	[2.70,3.62]	[2.78,3.68]	[2.70,3.62]
**Sex**				
Women	Ref.	Ref.	Ref.	Ref.
Men	1.27***	1.27***	1.44***	1.31***
	[1.13,1.41]	[1.13,1.42]	[1.30,1.57]	[1.17,1.45]
Constant	-2.53***	-2.55***	-1.61***	-2.60***
	[-2.80,-2.26]	[-2.82,-2.28]	[-1.83,-1.39]	[-2.87,-2.33]
Observations	6081	6081	6081	6081
BIC	6605	6580	7093	6580

**Table 6:** Generalized linear models developed for studying the association between ABSI, BMI and high blood glucose.

	High blood glucose OR glucose-lowering drugs			
	D1	D2	D3	D4
**ABSI**				
ABSI (SDS NHANES)	0.33**	0.17***	0.17***	
	[0.10,0.56]	[0.09,0.24]	[0.10,0.25]	
**BMI classes**				
Normal	Ref.	Ref.		Ref.
Overweight	0.55***	0.57***		0.58***
	[0.34,0.75]	[0.36,0.77]		[0.38,0.79]
Obesity 1	1.05***	1.08***		1.10***
	[0.84,1.27]	[0.87,1.29]		[0.89,1.31]
Obesity 2 and 3	1.57***	1.63***		1.64***
	[1.33,1.81]	[1.40,1.87]		[1.40,1.87]
**BMI-ABSI interaction**				
Normal # ABSI (SDS NHANES)	Ref.			
Overweight # ABSI (SDS NHANES)	-0.06			
	[-0.32,0.20]			
Obesity 1 # ABSI (SDS NHANES)	-0.22			
	[-0.49,0.04]			
Obesity 2 and 3 # ABSI (SDS NHANES)	-0.38*			
	[-0.67,-0.08]			
**Age classes**				
19 to 29 years	Ref.	Ref.	Ref.	Ref.
30 to 39 years	0.51**	0.52**	0.51**	0.53**
	[0.16,0.87]	[0.16,0.88]	[0.15,0.86]	[0.18,0.89]
40 to 49 years	1.17***	1.18***	1.18***	1.20***
	[0.83,1.50]	[0.84,1.51]	[0.85,1.51]	[0.86,1.53]
50 to 59 years	1.81***	1.82***	1.85***	1.84***
	[1.48,2.15]	[1.49,2.16]	[1.52,2.18]	[1.51,2.17]
60 to 69 years	2.07***	2.09***	2.20***	2.10***
	[1.72,2.41]	[1.74,2.43]	[1.86,2.54]	[1.75,2.44]
70 to 76 years	2.19***	2.20***	2.33***	2.20***
	[1.77,2.61]	[1.78,2.62]	[1.91,2.74]	[1.78,2.62]
**Sex**				
Women	Ref.	Ref.	Ref.	Ref.
Men	0.91***	0.89***	1.05***	0.94***
	[0.78,1.04]	[0.76,1.03]	[0.92,1.17]	[0.81,1.07]
Constant	-3.30***	-3.33***	-2.61***	-3.39***
	[-3.66,-2.94]	[-3.69,-2.98]	[-2.93,-2.30]	[-3.75,-3.04]
Observations	6081	6081	6081	6081
BIC	6343	6328	6547	6337

Values are regression coefficients, 95% confidence intervals in brackets and BIC.

Model 1: ABSI (continuous), BMI (discrete), ABSI·BMI interaction (continuous X discrete), sex (discrete), age (discrete).

Model 2: same as #1 without the ABSI·BMI interaction.

Model 3: same as #2 without BMI.

Model 4: same as #2 without ABSI.

Abbreviations: Ref = Reference category (regression coefficient = 0)

* p<0.05

** p<0.01

*** p<0.001

**S1–S4 Figs** give the effects plots corresponding to models A2, B1, C2 and D1. These figures show the joint effects of BMI and ABSI taking into account their interaction when significant.

**Table 7**gives the regression coefficients and AIC for the 4 GLM used to investigate the association between VAT and ABSI. ABSI and BMI contributed independently to VAT (model E1) and there was a clear ABSI·BMI interaction (model E1). Compared to BMI alone, the joint use of BMI and ABSI was more strongly associated with VAT (AIC = 22788 for model E1 vs. 23412 for model E2).

**Table 7:** Generalized linear models developed for studying the association between ABSI, BMI and VAT.

	VAT			
	E1	E2	E3	E4
**ABSI**				
ABSI (SDS NHANES)	0.24***	0.62***	0.65***	
	[0.13,0.35]	[0.57,0.67]	[0.59,0.72]	
**BMI classes**				
Normal weight	Ref.	Ref.		Ref.
Overweight	1.07***	0.95***		1.02***
	[0.96,1.19]	[0.83,1.06]		[0.91,1.14]
Obesity 1	2.49***	2.34***		2.44***
	[2.37,2.62]	[2.22,2.46]		[2.32,2.57]
Obesity 2 and 3	4.50***	4.34***		4.38***
	[4.35,4.65]	[4.19,4.48]		[4.22,4.53]
**BMI-ABSI interaction**				
Normal # ABSI (SDS NHANES)	Ref.			
Overweight # ABSI (SDS NHANES)	0.35***			
	[0.22,0.49]			
Obesity 1 # ABSI (SDS NHANES)	0.61***			
	[0.46,0.75]			
Obesity 2 and 3 # ABSI (SDS NHANES)	0.55***			
	[0.38,0.73]			
**Age classes**				
19 to 29 years	Ref.	Ref.	Ref.	Ref.
30 to 39 years	0.31***	0.28***	0.32**	0.39***
	[0.15,0.47]	[0.11,0.44]	[0.11,0.53]	[0.22,0.56]
40 to 49 years	0.81***	0.76***	0.90***	0.93***
	[0.65,0.96]	[0.61,0.92]	[0.70,1.10]	[0.77,1.09]
50 to 59 years	1.33***	1.29***	1.57***	1.47***
	[1.17,1.49]	[1.13,1.45]	[1.37,1.78]	[1.30,1.64]
60 to 69 years	1.88***	1.82***	2.35***	1.95***
	[1.70,2.05]	[1.64,1.99]	[2.13,2.57]	[1.77,2.14]
70 to 76 years	2.38***	2.33***	2.87***	2.43***
	[2.14,2.63]	[2.08,2.57]	[2.55,3.19]	[2.17,2.69]
**Sex**				
Women	Ref.	Ref.	Ref.	Ref.
Men	2.05***	2.08***	2.58***	2.29***
	[1.96,2.15]	[1.99,2.18]	[2.46,2.69]	[2.19,2.38]
Constant	2.33***	2.49***	3.71***	2.13***
	[2.17,2.49]	[2.33,2.64]	[3.53,3.89]	[1.97,2.29]
Observations	6081	6081	6081	6081
BIC	22882	22930	26044	23479

Values are regression coefficients, 95% confidence intervals in brackets and BIC.

Model 1: ABSI (continuous), BMI (discrete), ABSI·BMI interaction (continuous X discrete), sex (discrete), age (discrete).

Model 2: same as #1 without the ABSI·BMI interaction.

Model 3: same as #2 without BMI.

Model 4: same as #2 without ABSI.* p<0.05

** p<0.01

*** p<0.001

**S5 Fig** gives the effects plots corresponding to model E1. This figure shows the joint effects of BMI and ABSI taking into account their interaction.

We also conducted a supplementary analysis restricted to subjects who were not following a drug therapy for controlling cardio-metabolic risk factors (n = 4800). The associations of BMI and ABSI with each cardio-metabolic risk factor remained very similar (**S1 Table**).

The sign of the interaction between ABSI and high BMI category was negative for low HDL and high fasting glucose, implying that a given increment of ABSI increases risk relatively more for less heavy people (Tables 4 and 6, S2 and S4 Figs). By contrast, the interaction coefficient was positive for VAT, implying that high ABSI affects VAT more when BMI is also high (Table 7, S5 Fig).

## Discussion

In the present study, we tested the separate and the joint contribution of ABSI and BMI to high triglycerides, low HDL, high blood pressure, high fasting glucose and VAT, taking into account the effects of sex and age. We found that ABSI was independently associated with all outcomes. Moreover, the joint use of BMI and ABSI was associated with a better estimate of the probability of having high triglycerides, low HDL and high fasting glucose and of VAT thickness as compared to BMI or ABSI alone. Interestingly, an ABSI·BMI interaction was detected for low HDL, high fasting glucose and VAT. These findings did not change when we restricted the analysis to subjects who were not following a drug therapy for controlling the cardio-metabolic risk factors.

The greatest strength of this study is that it was performed at a single clinical research center in a very large population of subjects with strictly standardized methods. Another strength of this study is that the large number of enrolled subjects allowed to estimate relatively precisely the degree of statistical interaction between ABSI and BMI and therefore to assess whether these anthropometric measures associate independently with CMD risk factors.

Among the study limitations are the following. First, we studied a self-selected sample of Caucasian adults and our findings are not necessarily applicable to other populations. Second, we measured WC at the WHO landmark and not at the NHANES landmark. Although the WC measures obtained at the WHO and NHANES landmarks are well correlated, this may have produced some misclassification of ABSI [35]. Third, VAT was measured by US, which is not a reference method. However, the US measurement procedure has been thoroughly validated against computed tomography [29, 36] and used in previous studies [25, 30, 31, 37].

WC, a surrogate measure of abdominal adiposity, is an established risk factor for CMD, but its strong association with BMI makes it difficult to assess its independent contribution to CMD [1, 12]. By standardizing WC on its value estimated from allometric regression of weight and height, ABSI offers a WC measure which is independent from weight and BMI, allowing the joint use of BMI and WC to study CMD [1, 38, 39]. Thus, ABSI has great potential for being used in place of WC and together with BMI to evaluate the independent contributions of body mass and shape on CVD [10, 11].

In a cross-sectional study of subjects with type 2 diabetes mellitus, ABSI was associated with visceral fat area estimated by bioelectrical impedance analysis [17]. Using US to directly measure VAT thickness, the present study confirms that ABSI is directly associated with VAT and is thus a surrogate measure of abdominal adiposity. The association that we detected between ABSI and VAT supports the association ABSI to CMD risk factors. Such association might also partly explain the observed association of ABSI with overall mortality [1, 38, 39].

In a large cross-sectional study performed in Iran, BMI and the waist-to-height ratio were stronger than ABSI at predicting CVD risk factors [13]. One cross-sectional study and one cohort study reported a modest association between ABSI and hypertension and hypercholesterolemia [14, 15]. Unfortunately, these studies did not evaluate the joint contribution of ABSI and BMI to CMD risk factors. Our study confirms that ABSI taken alone is less accurate than BMI at detecting CMD risk factors, but it also shows that the joint use of ABSI and BMI is superior to BMI (and ABSI) to identify the occurrence of low HDL, high triglycerides and high glucose.

## Conclusion

In conclusion, ABSI is associated with all the components of the MS and with VAT thickness. The joint use of ABSI and BMI allows a better assessment of the probability of low HDL, high triglycerides and high fasting glucose and of VAT thickness as compared to BMI alone. ABSI can be a useful index, as opposed to WC, for evaluating the relative contribution of central obesity to clinical outcomes in complement with and not as an alternative to BMI. Further studies aimed to evaluate the capability of ABSI-BMI to jointly predict longitudinal outcomes are warranted.

## Supporting information

S1 Fig Marginal probabilities of having high triglycerides obtained from model A2. The values of ABSI correspond to the internal 5^th^, 25^th^, 50^th^, 75^th^ and 95^th^ percentiles. Values are probabilities and 95% confidence intervals.
(PDF)

S2 Fig Marginal probabilities of having low HDL obtained from model B1. The values of ABSI correspond to the internal 5^th^, 25^th^, 50^th^, 75^th^ and 95^th^ percentiles. Values are probabilities and 95% confidence intervals.
(PDF)

S3 Fig Marginal probabilities of having high blood pressure obtained from model C2. The values of ABSI correspond to the internal 5^th^, 25^th^, 50^th^, 75^th^ and 95^th^ percentiles. Values are probabilities and 95% confidence intervals.
(PDF)

S4 Fig Marginal probabilities of having high glucose obtained from model D1. The values of ABSI correspond to the internal 5^th^, 25^th^, 50^th^, 75^th^ and 95^th^ percentiles. Values are probabilities and 95% confidence intervals.
(PDF)

S5 Fig Marginal means of VAT obtained from model E1. The values of ABSI correspond to the internal 5^th^, 25^th^, 50^th^, 75^th^ and 95^th^ percentiles. Values are predicted means and 95% confidence intervals.
(PDF)

S1 Table Association between ABSI, BMI and metabolic syndrome components in subjects without drug therapy. (DOCX)
