# Supplementary figures and images for: Association of Body Shape Index (ABSI) with cardio-metabolic risk factors: A cross-sectional study of 6081 Caucasian adults
Source: PLoS One. 2017 Sep 25;12(9):e0185013. doi: 10.1371/journal.pone.0185013 (PMC5612697; doi:10.1371/journal.pone.0185013)

19 to 29 years

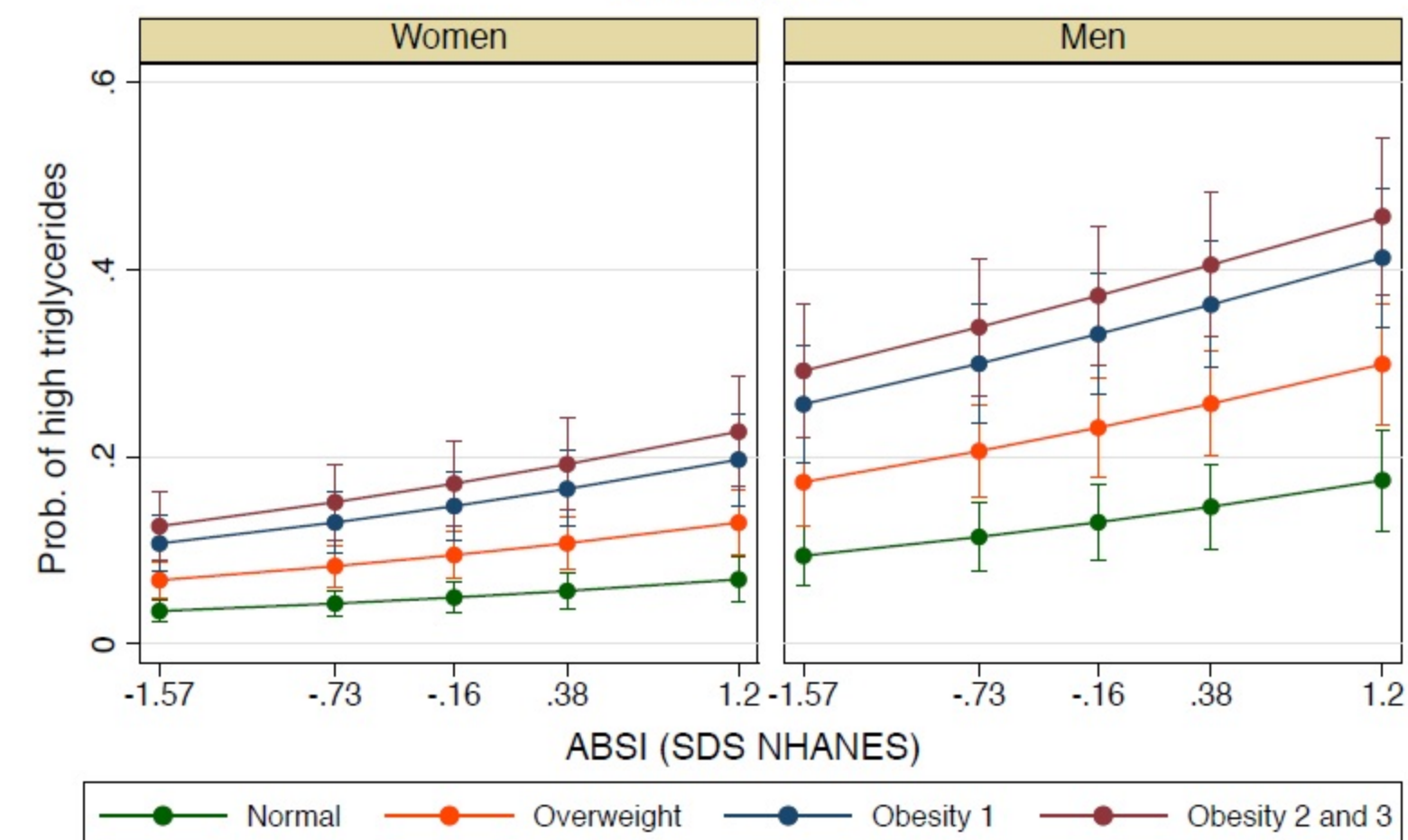

30 to 39 years

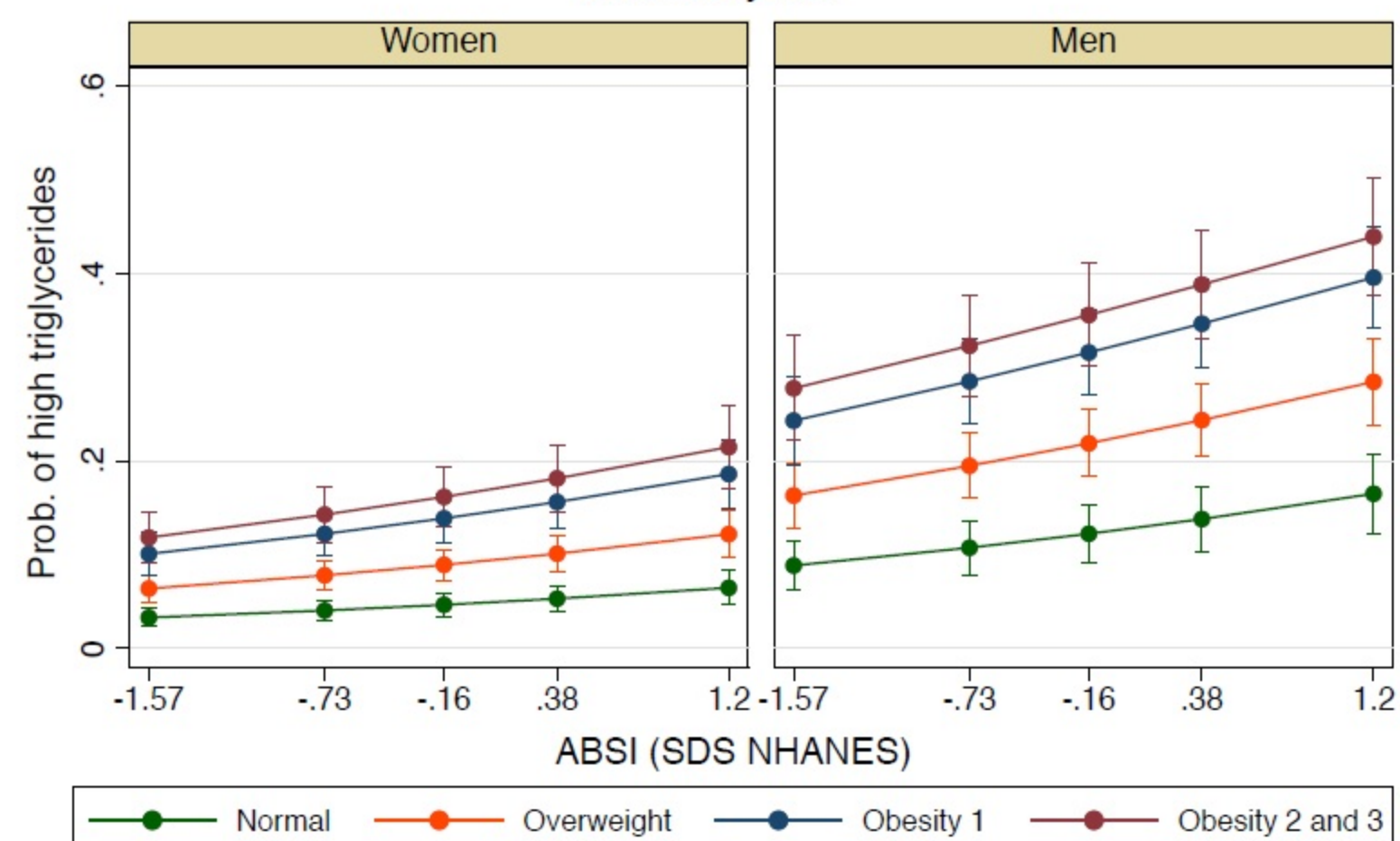

40 to 49 years

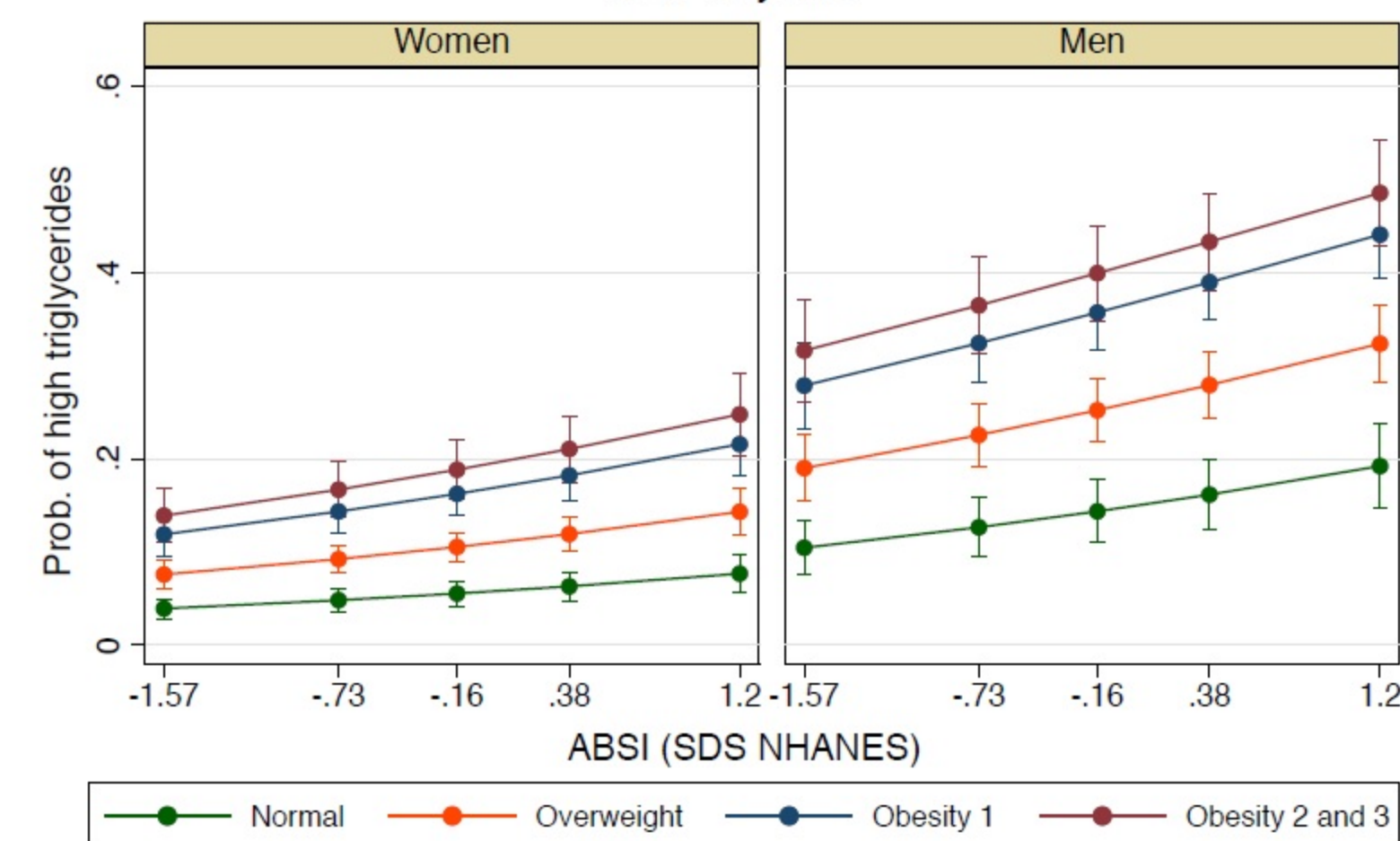

50 to 59 years

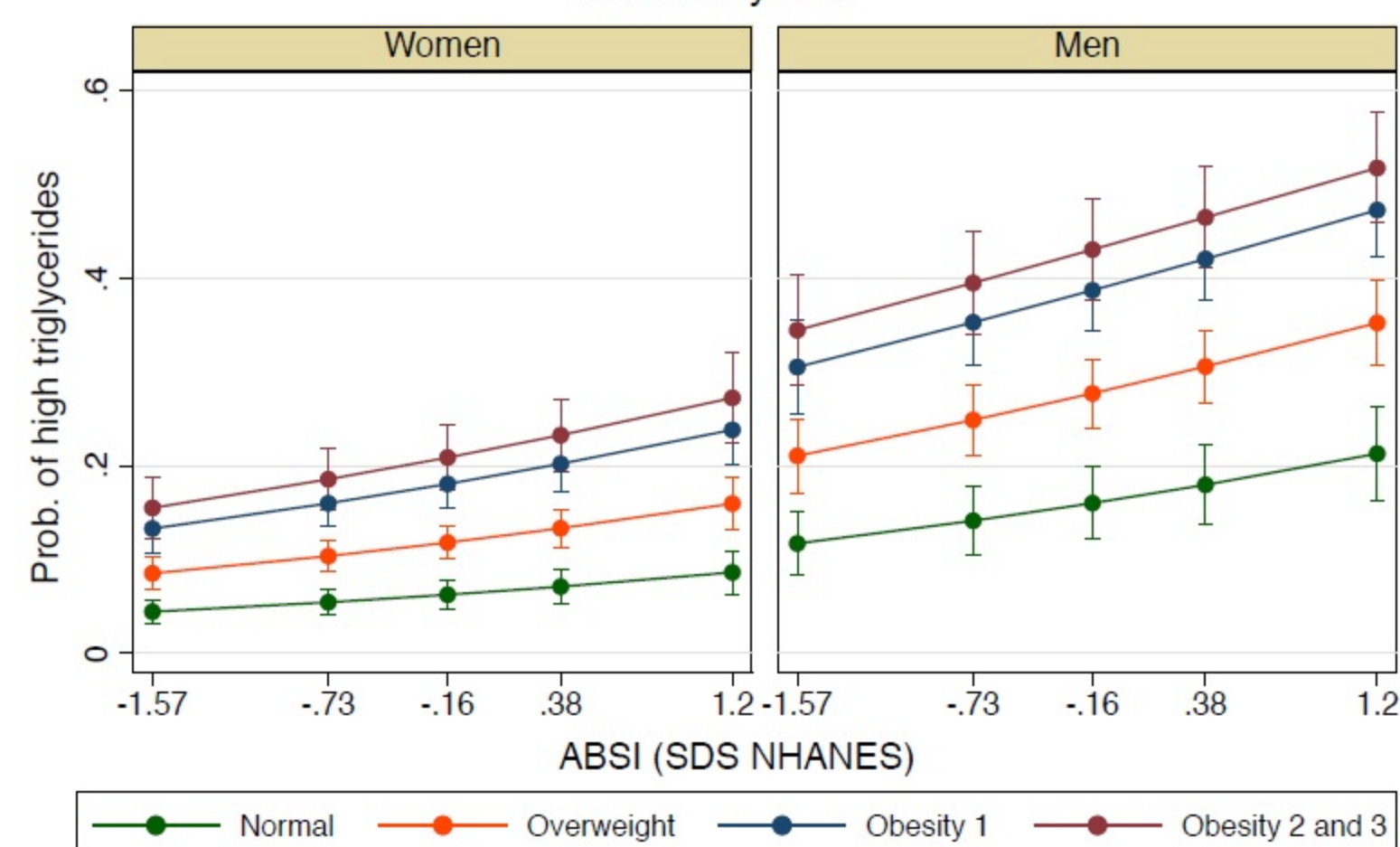

60 to 69 years

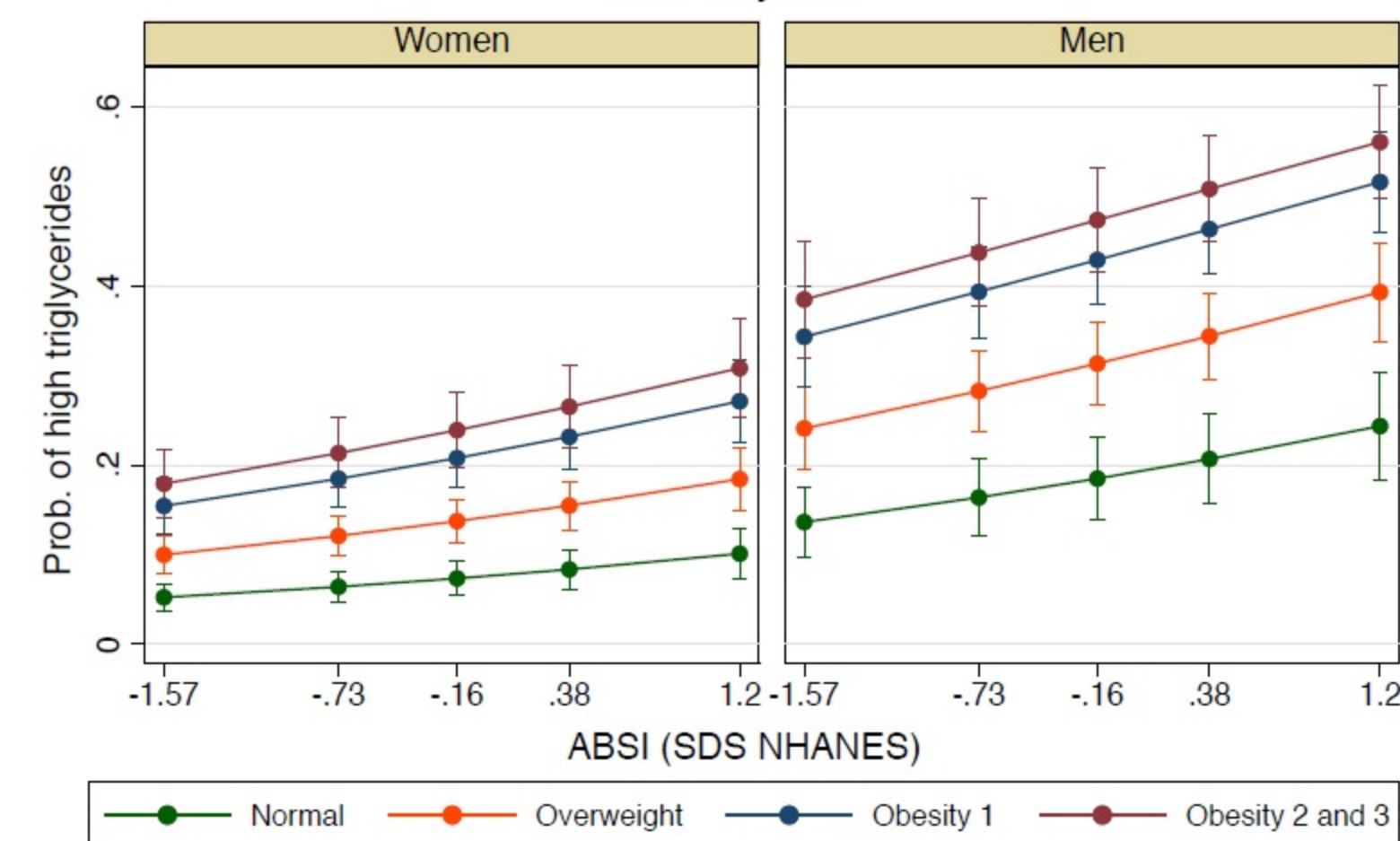

70 to 76 years

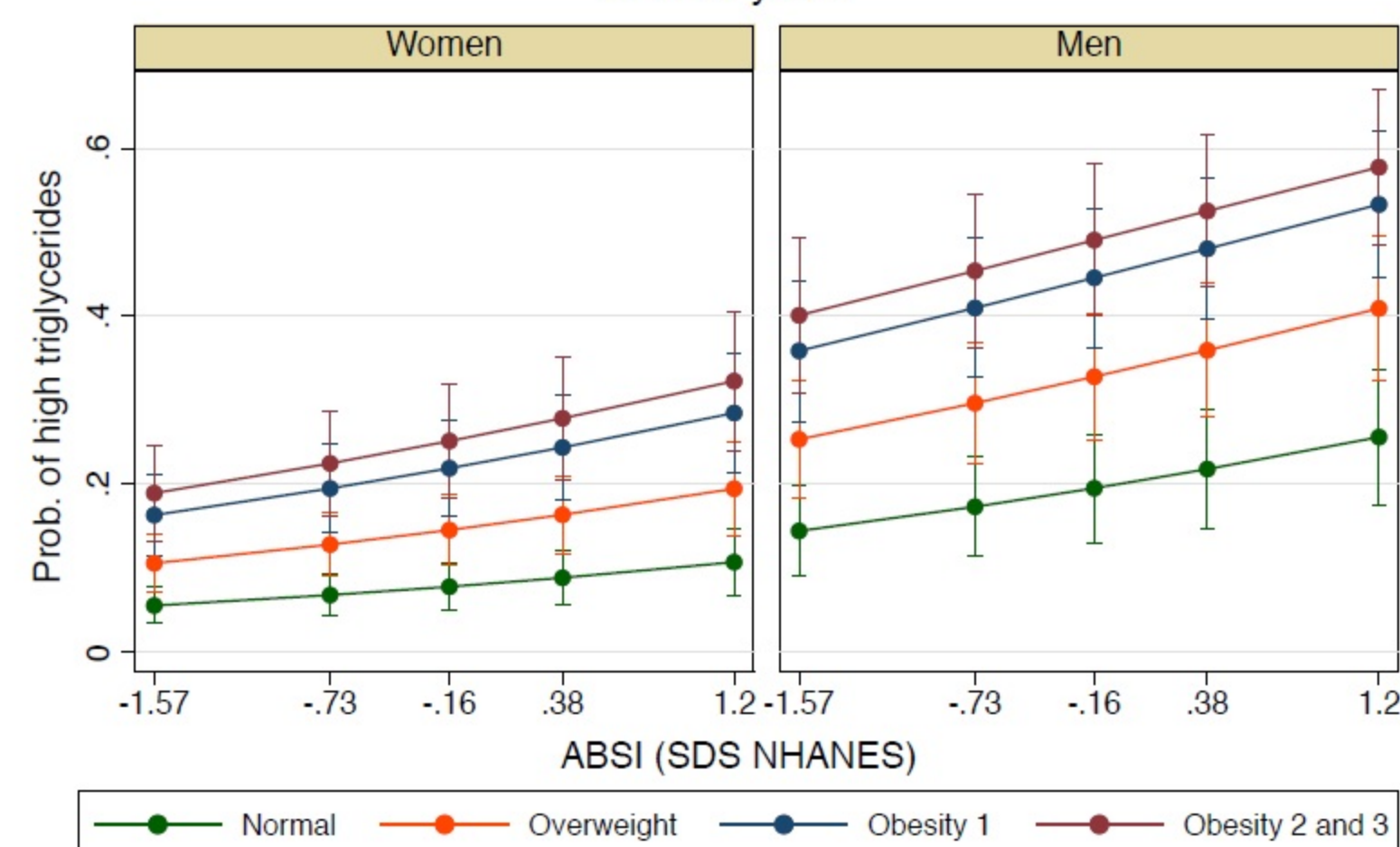

Supplement: S1 Fig — The values of ABSI correspond to the internal 5th, 25th, 50th, 75th and 95th percentiles. Values are probabilities and 95% confidence intervals. (PDF) [file pone.0185013.s001.pdf]

19 to 29 years

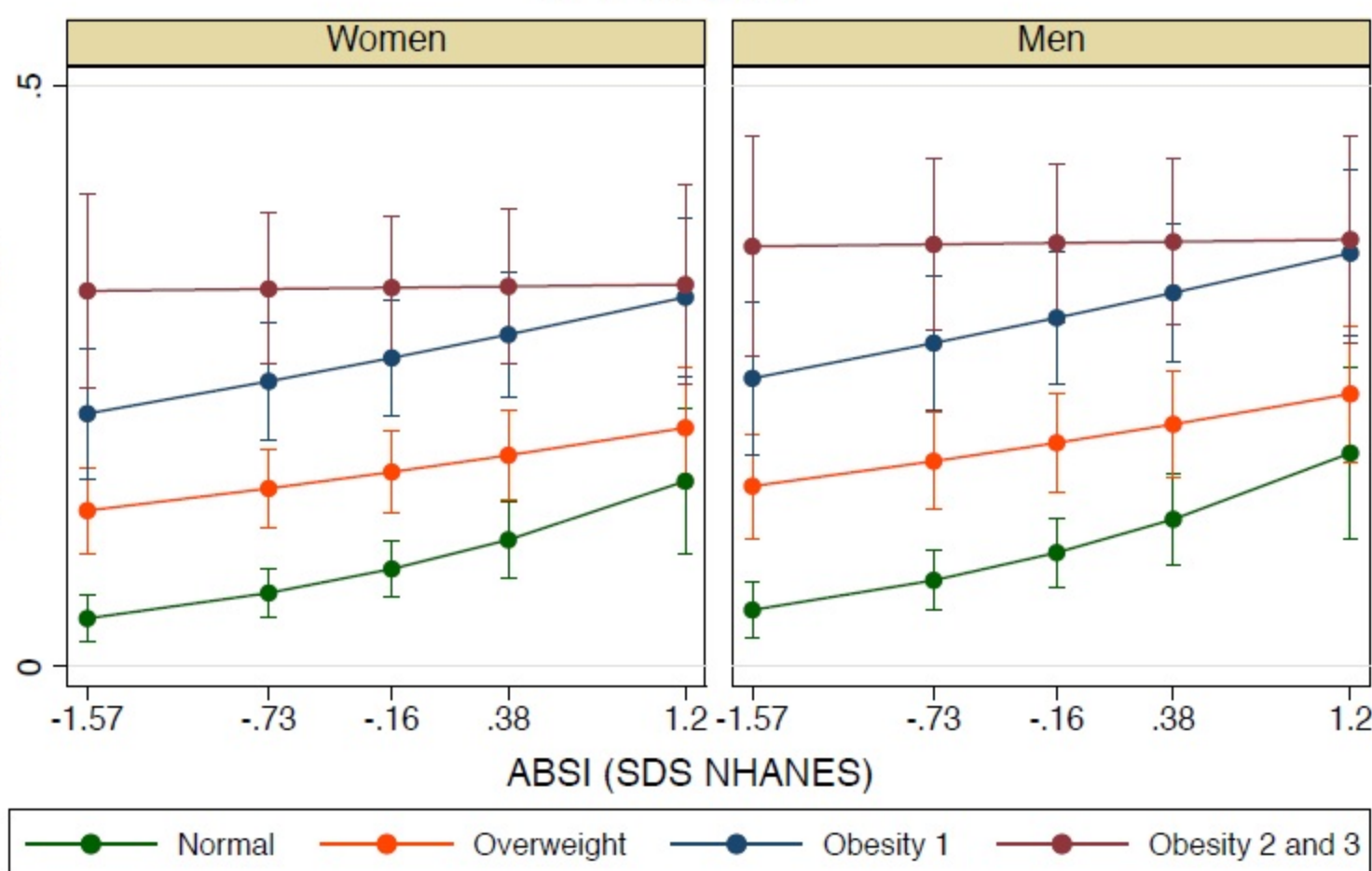

30 to 39 years

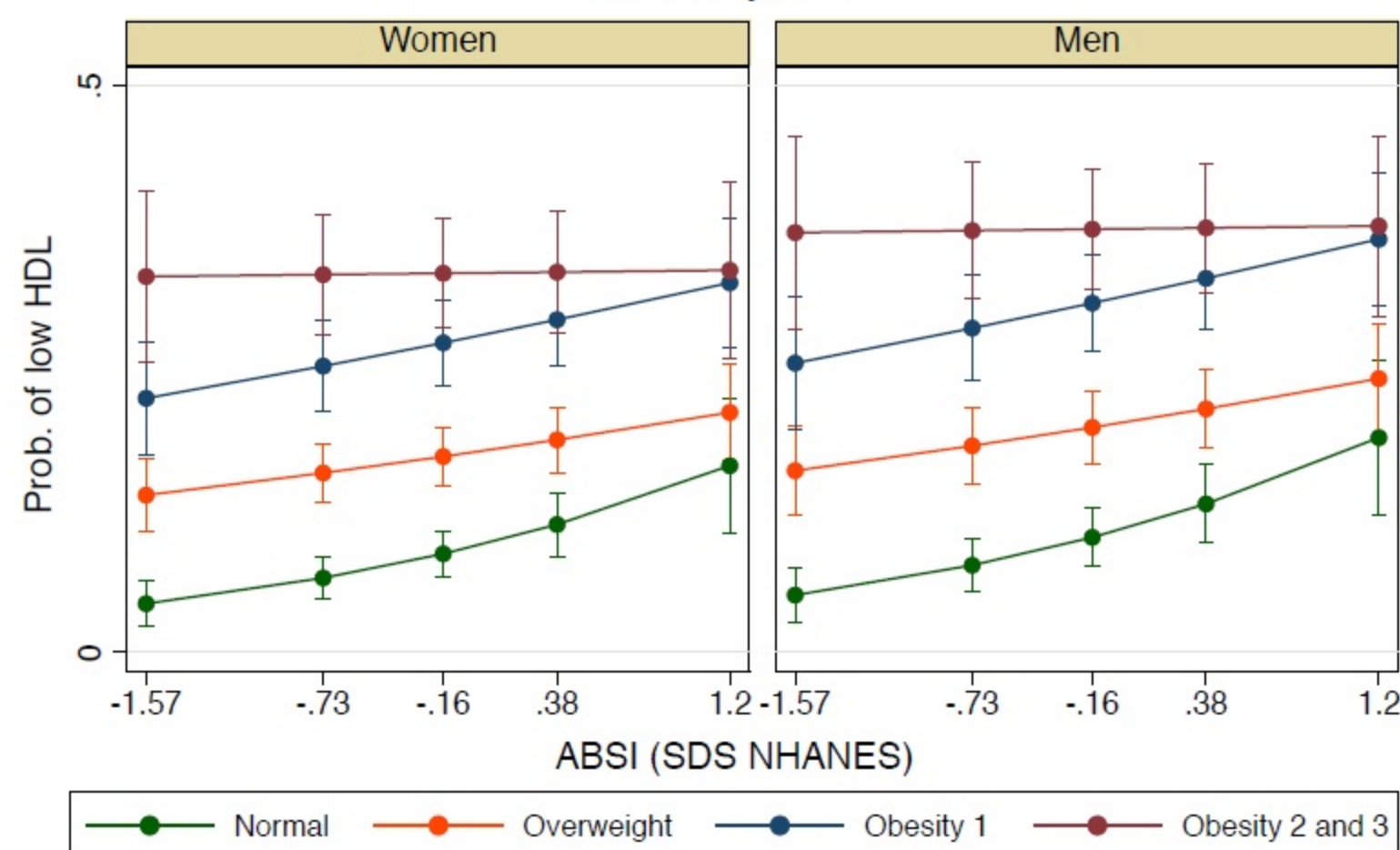

40 to 49 years

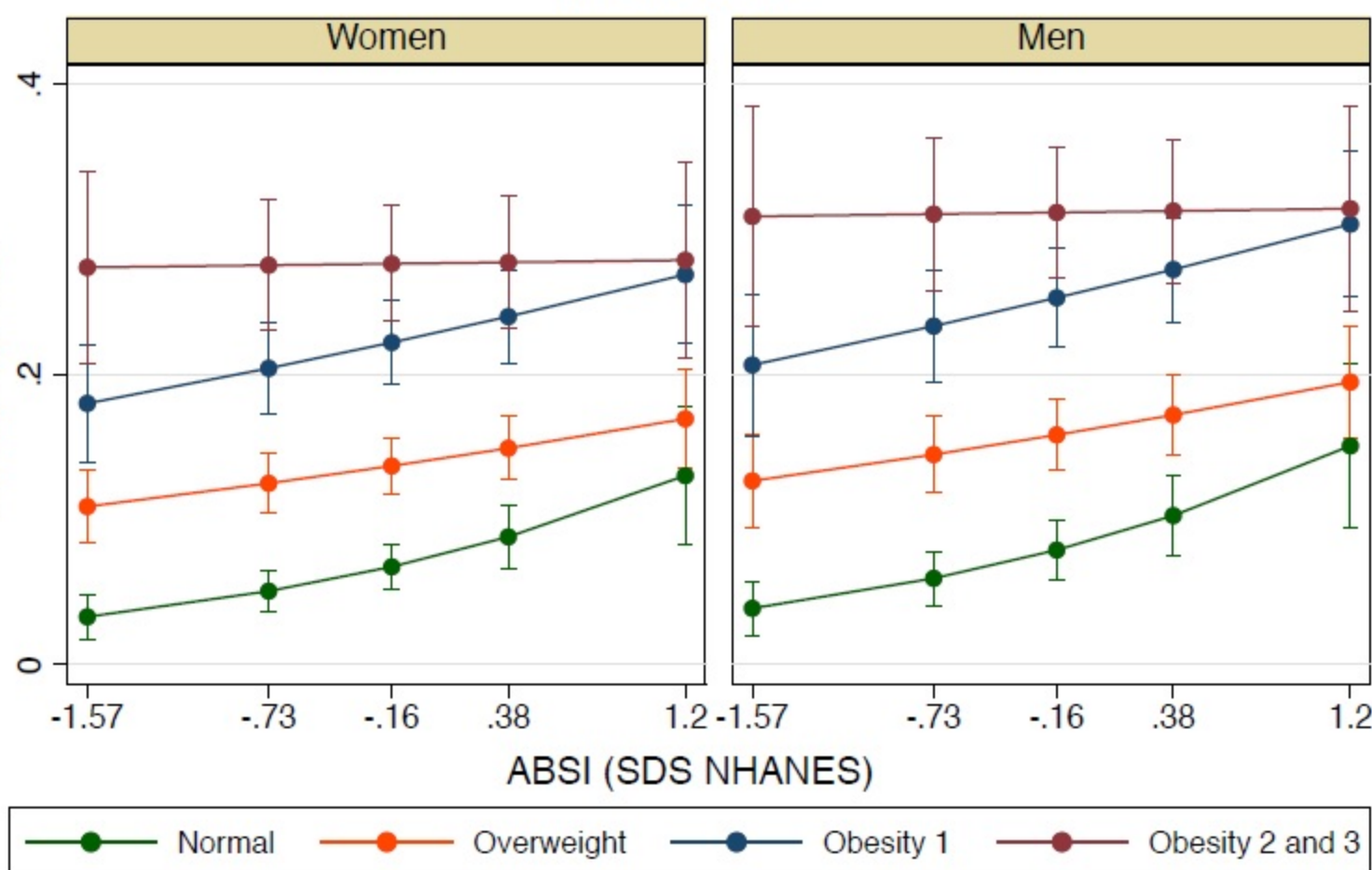

50 to 59 years

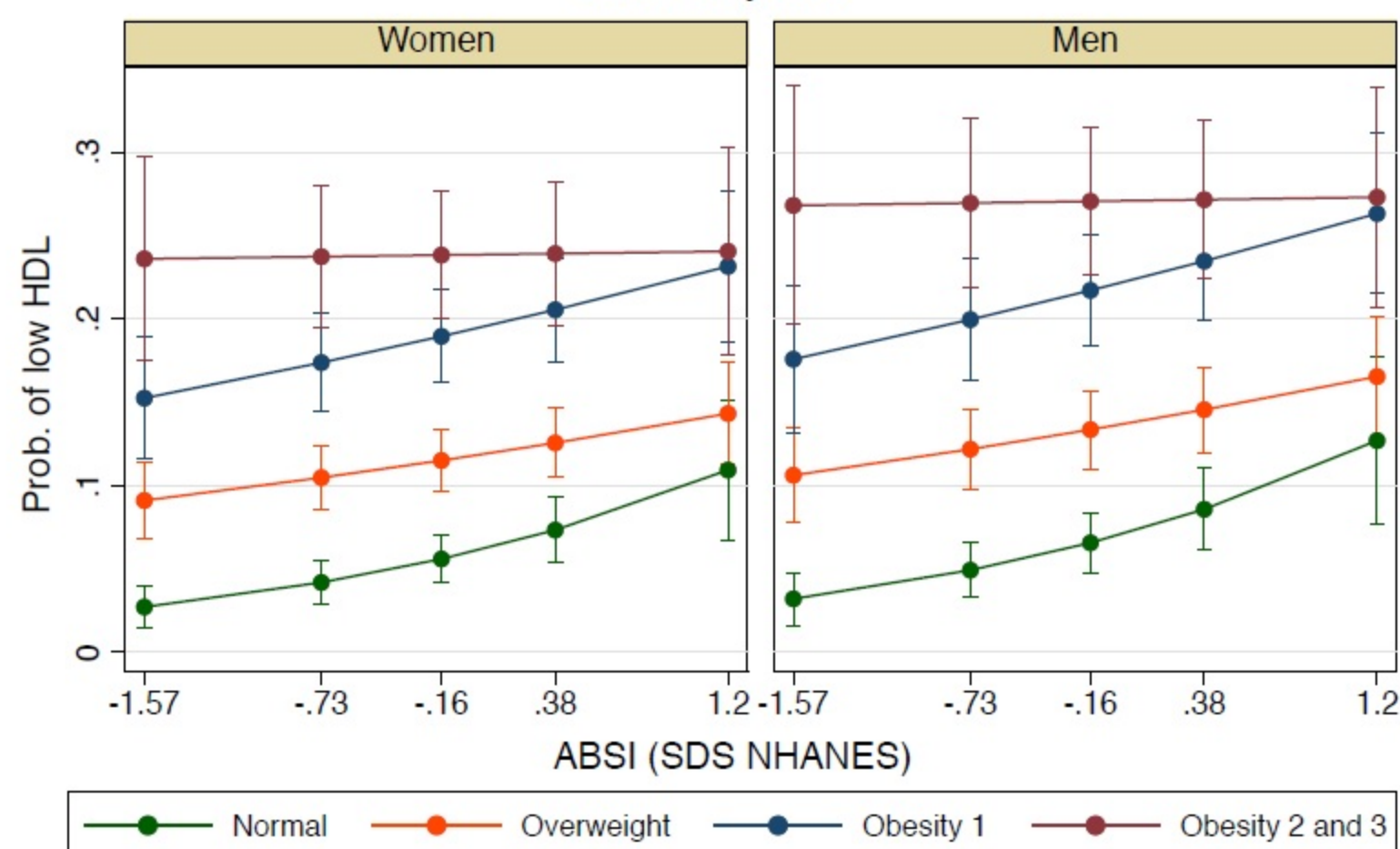

60 to 69 years

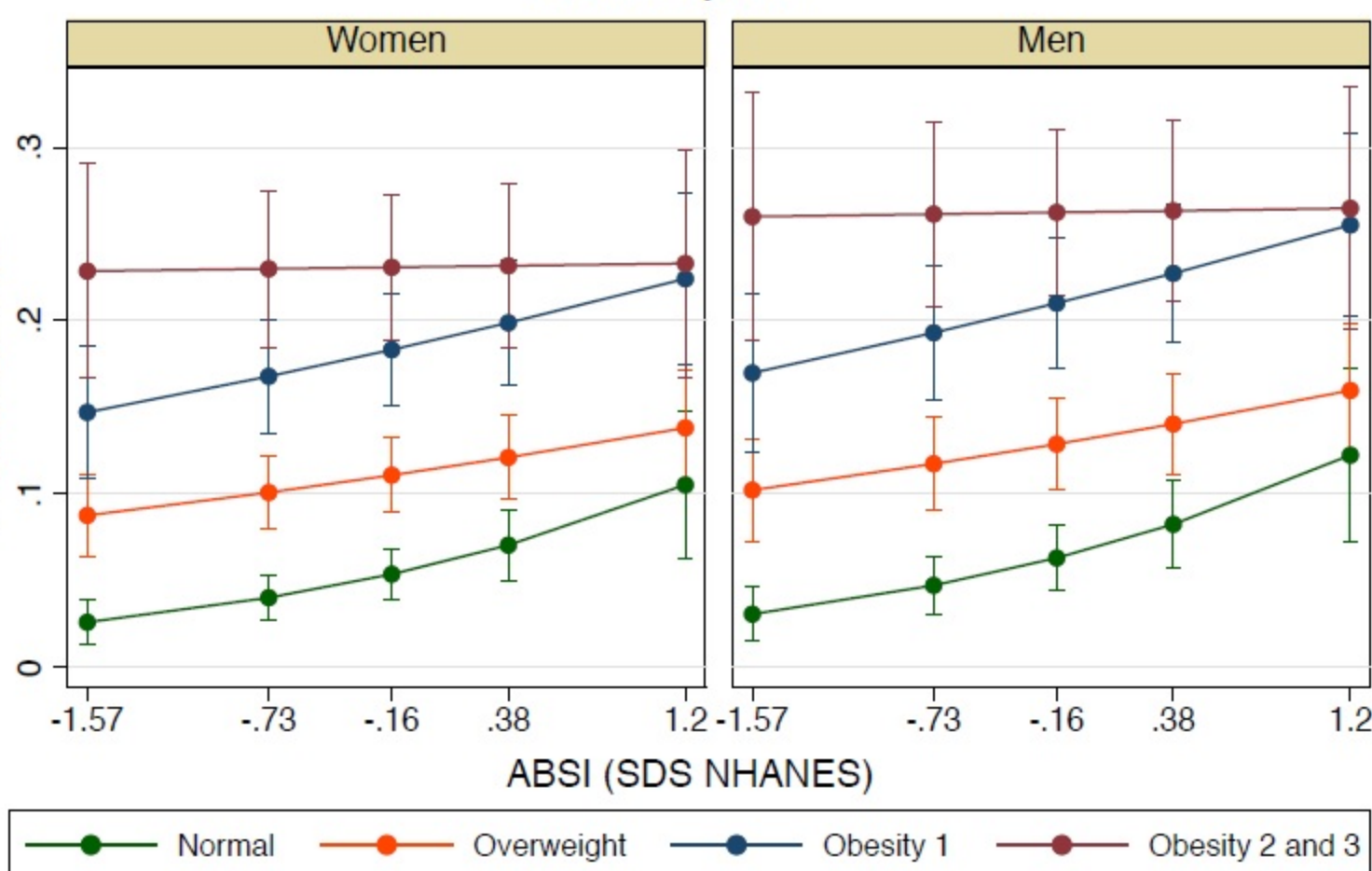

70 to 76 years

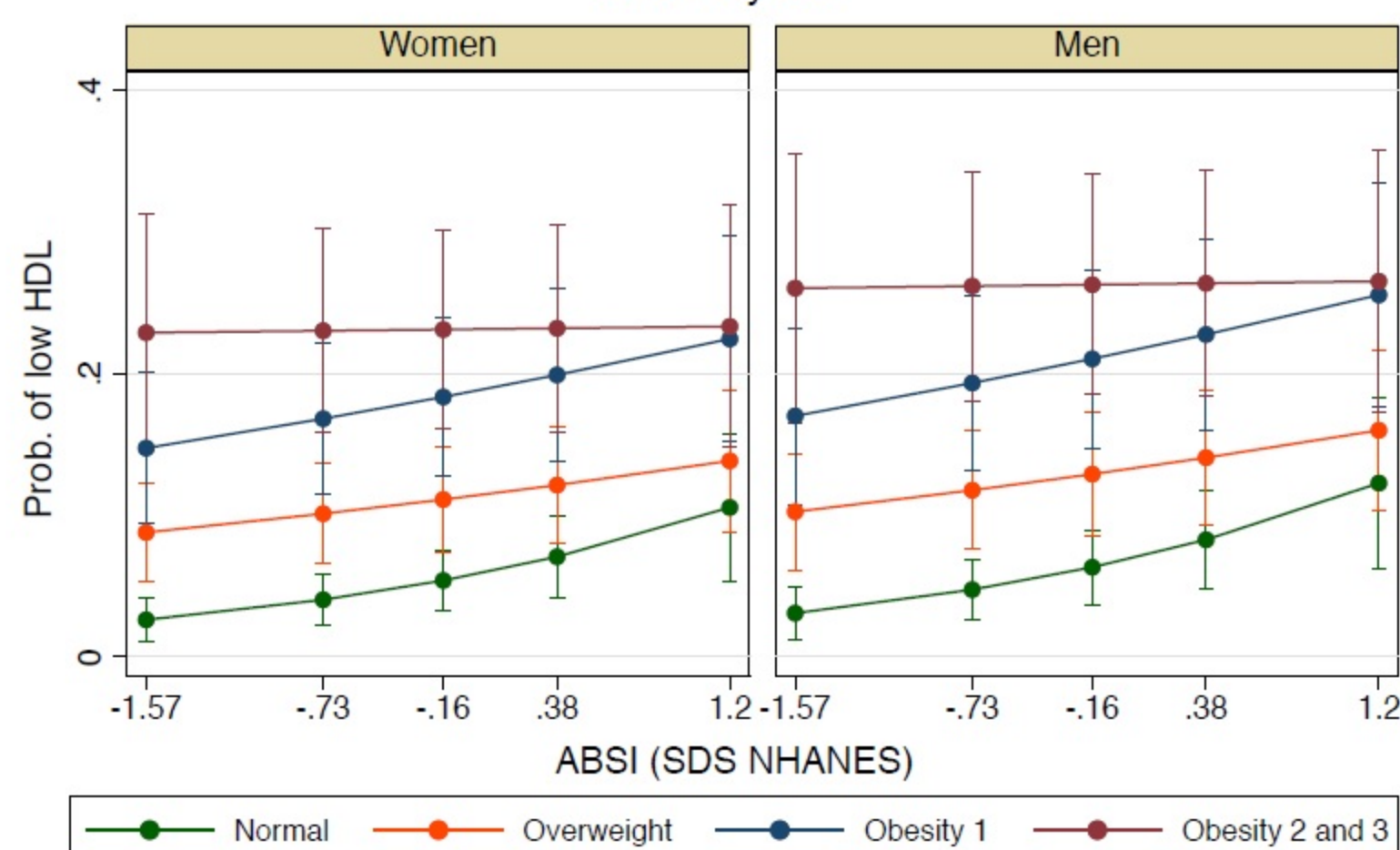

Supplement: S2 Fig — The values of ABSI correspond to the internal 5th, 25th, 50th, 75th and 95th percentiles. Values are probabilities and 95% confidence intervals. (PDF) [file pone.0185013.s002.pdf]

19 to 29 years

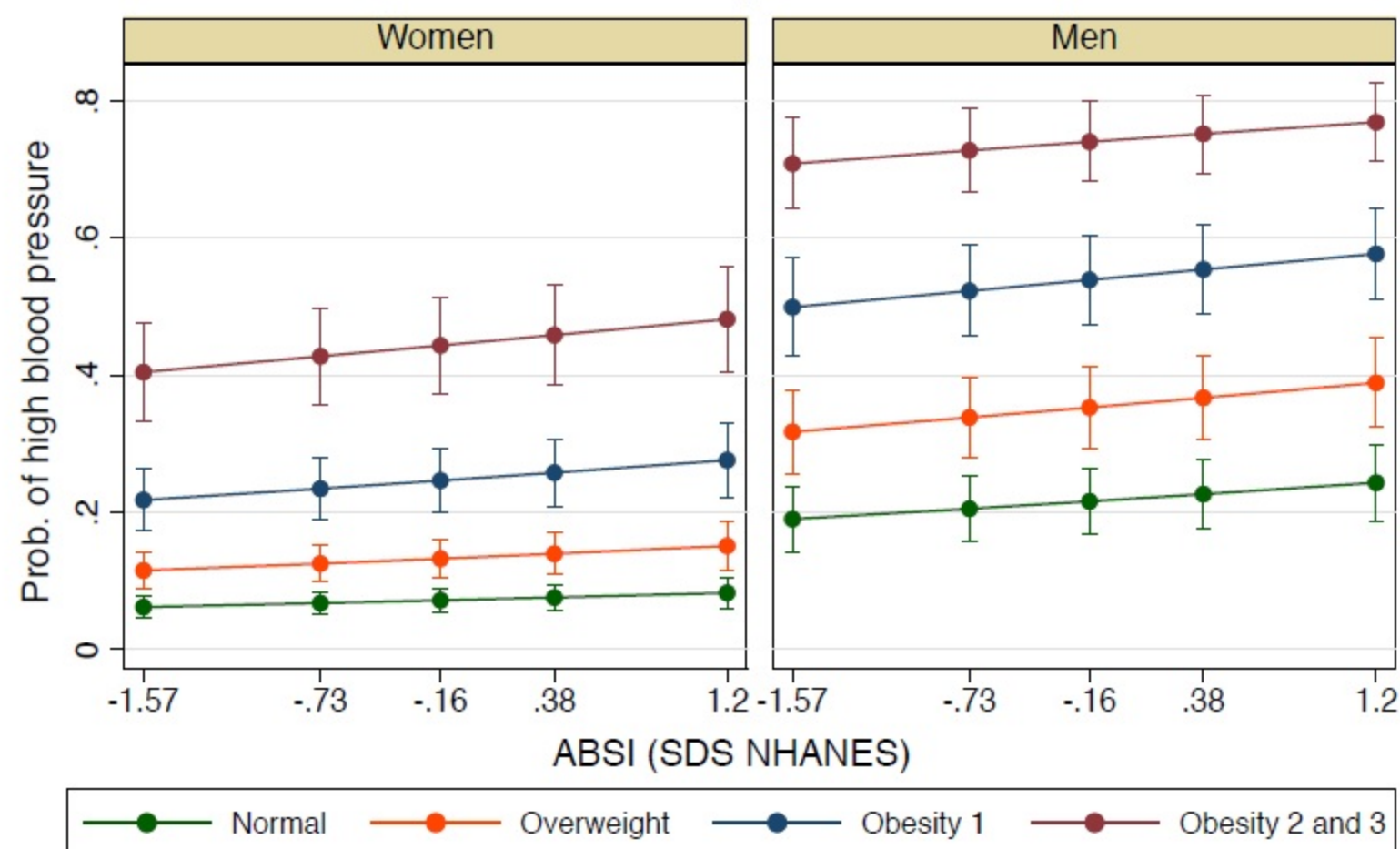

30 to 39 years

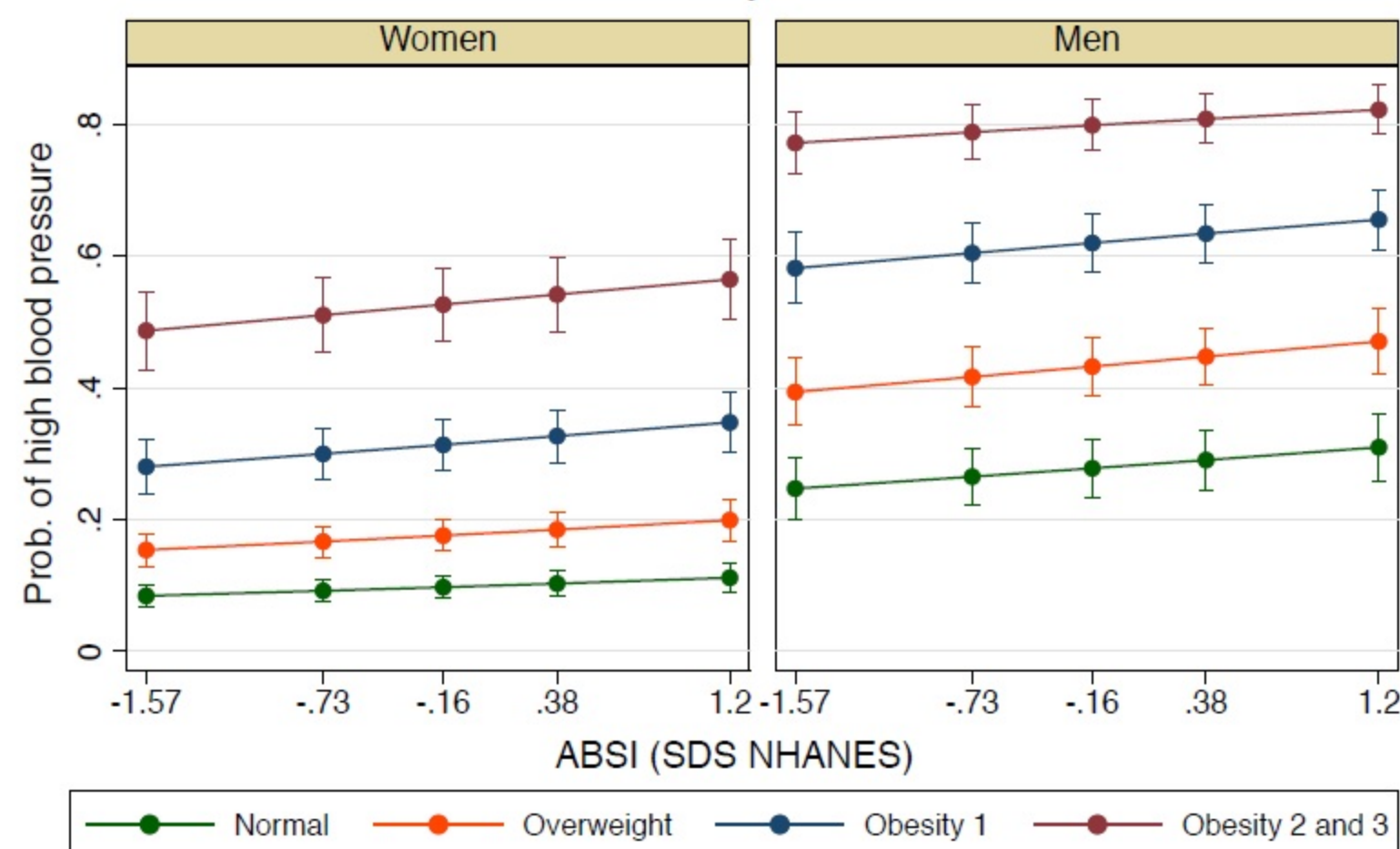

40 to 49 years

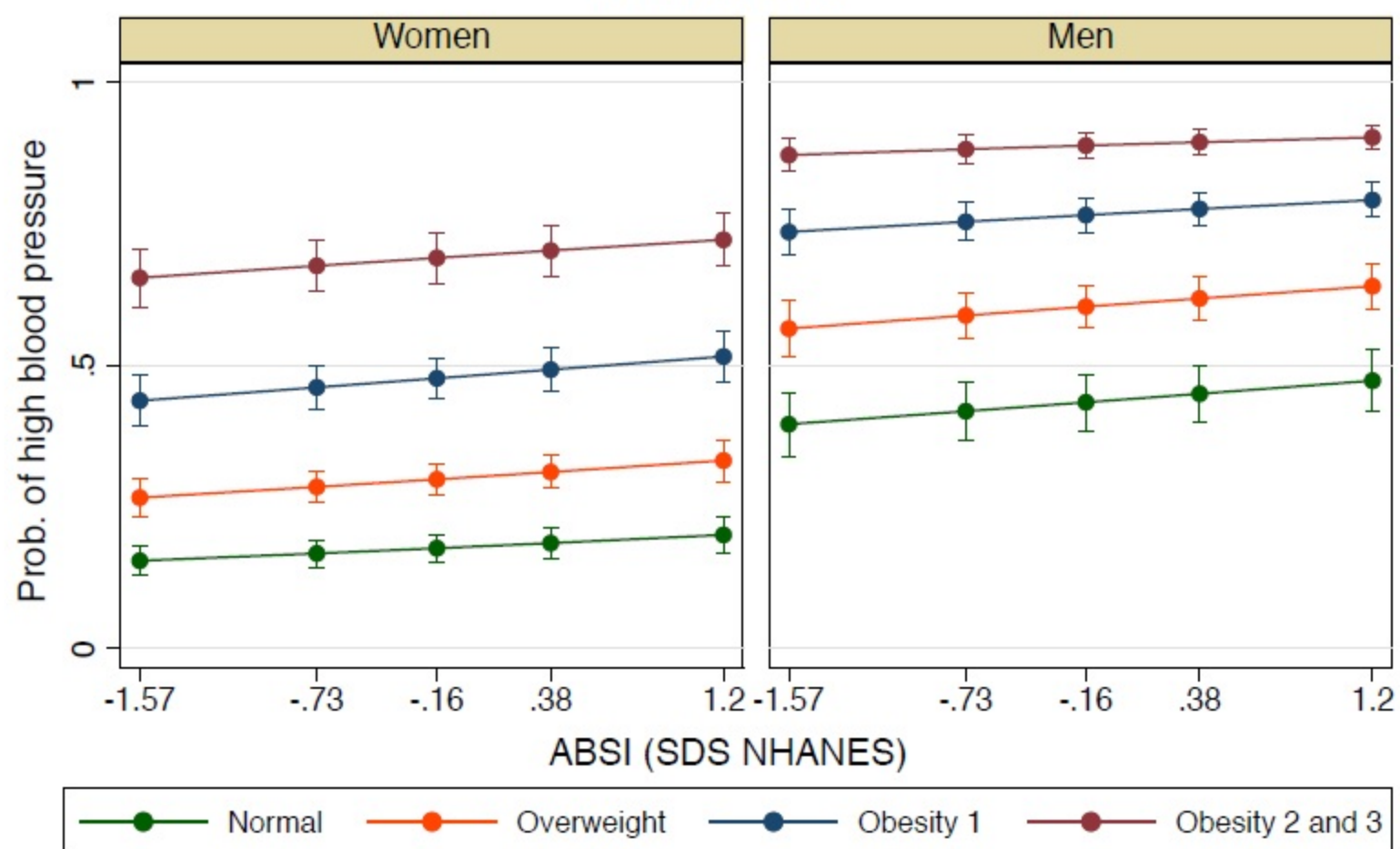

50 to 59 years

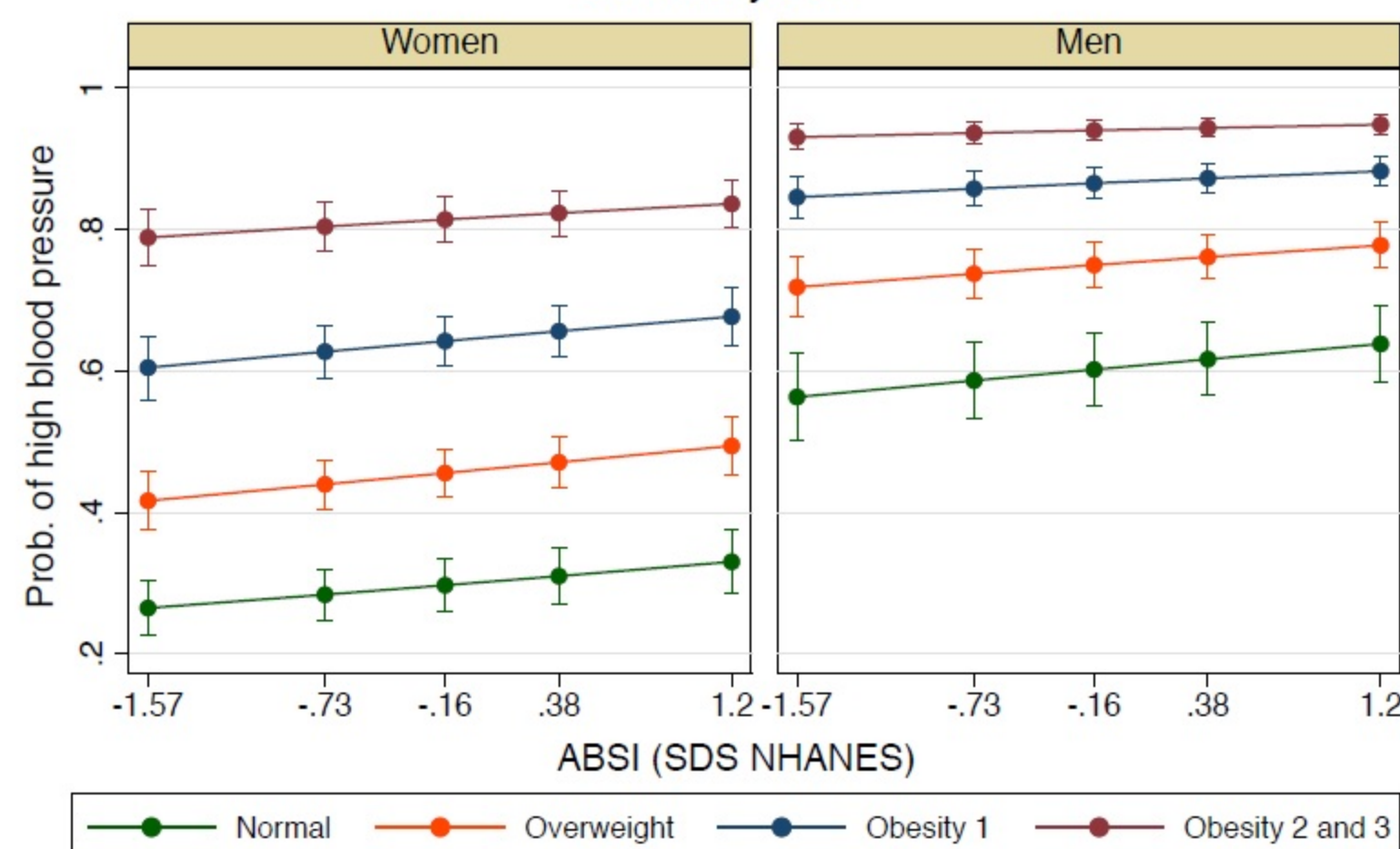

60 to 69 years

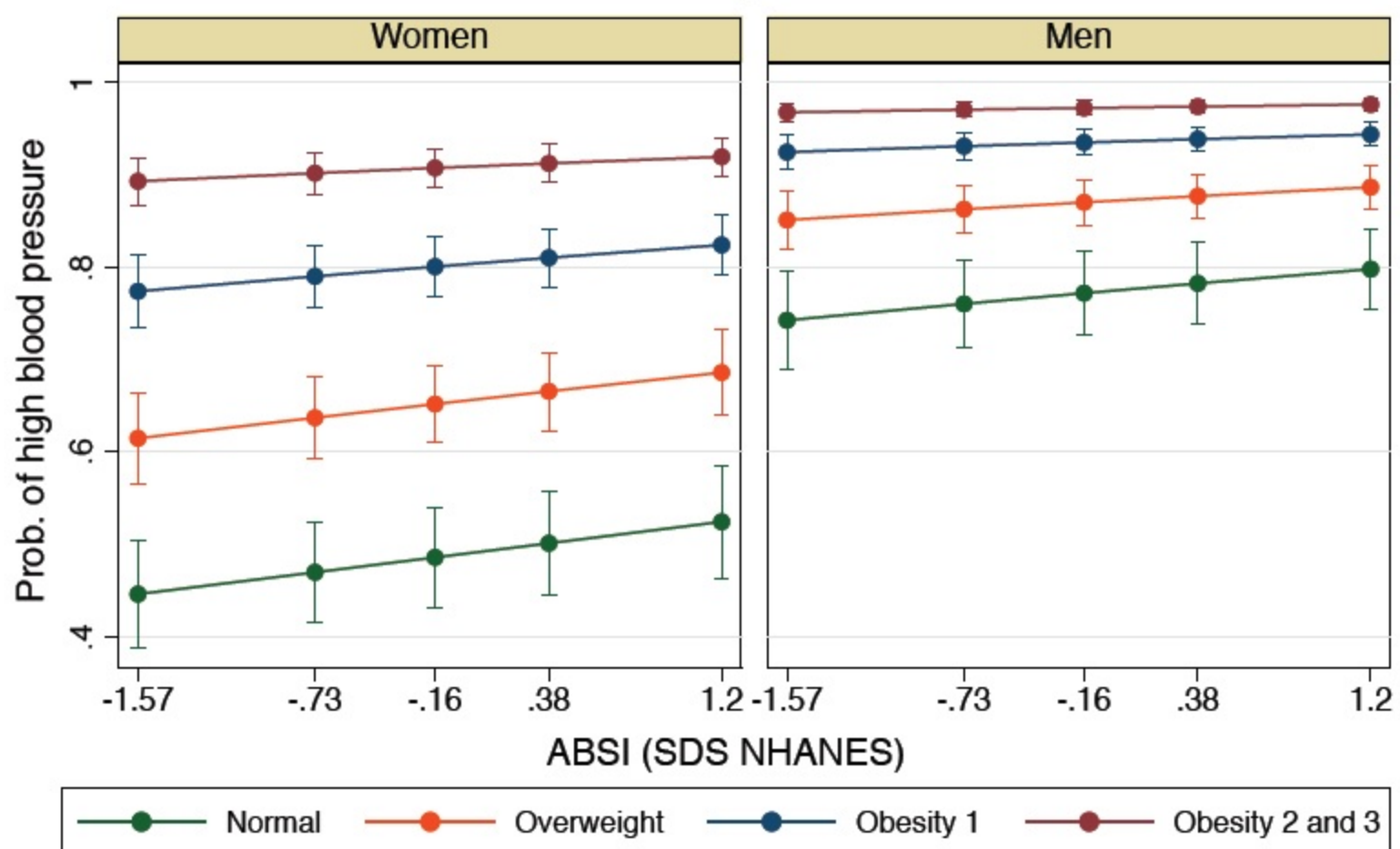

70 to 76 years

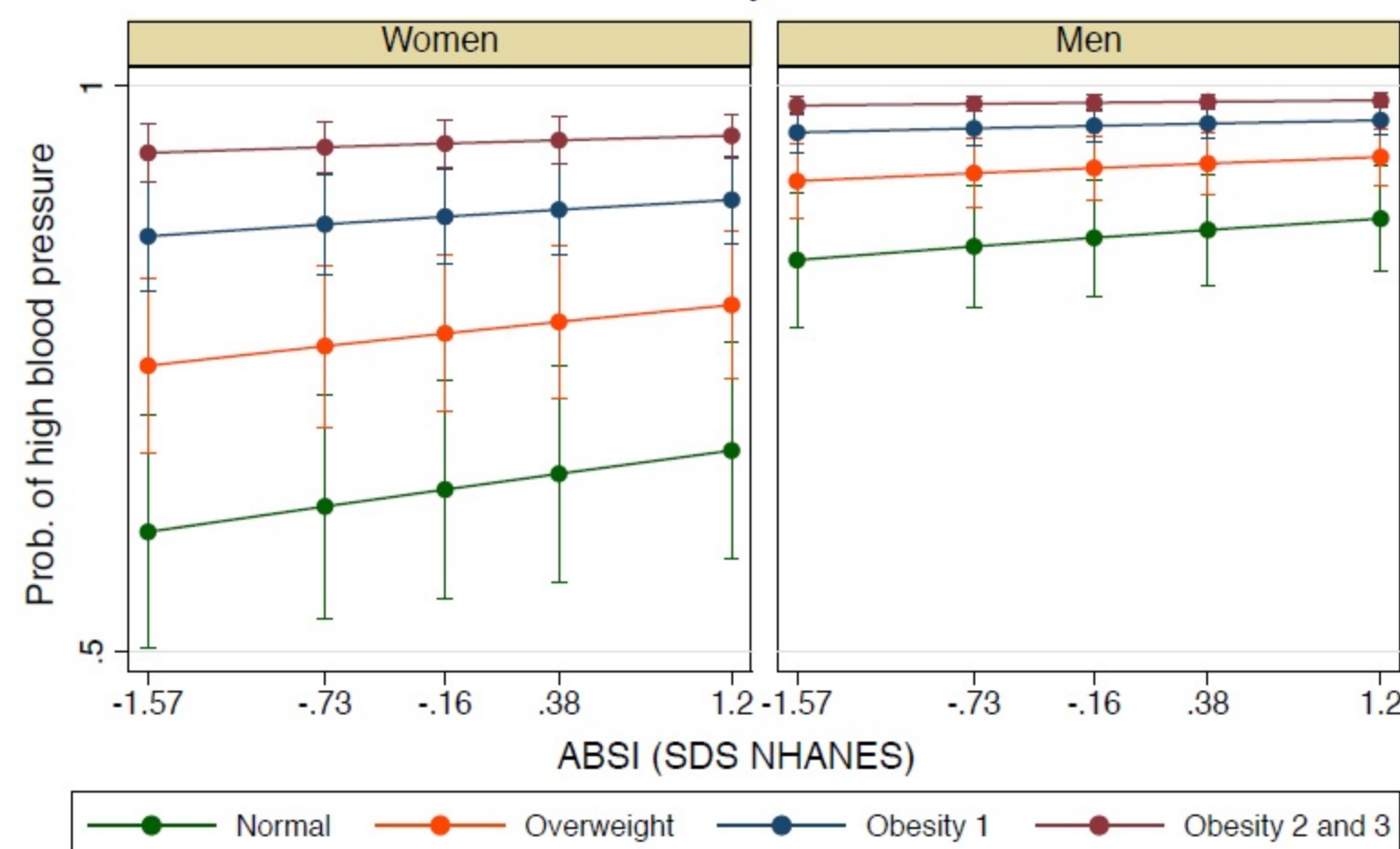

Supplement: S3 Fig — The values of ABSI correspond to the internal 5th, 25th, 50th, 75th and 95th percentiles. Values are probabilities and 95% confidence intervals. (PDF) [file pone.0185013.s003.pdf]

19 to 29 years

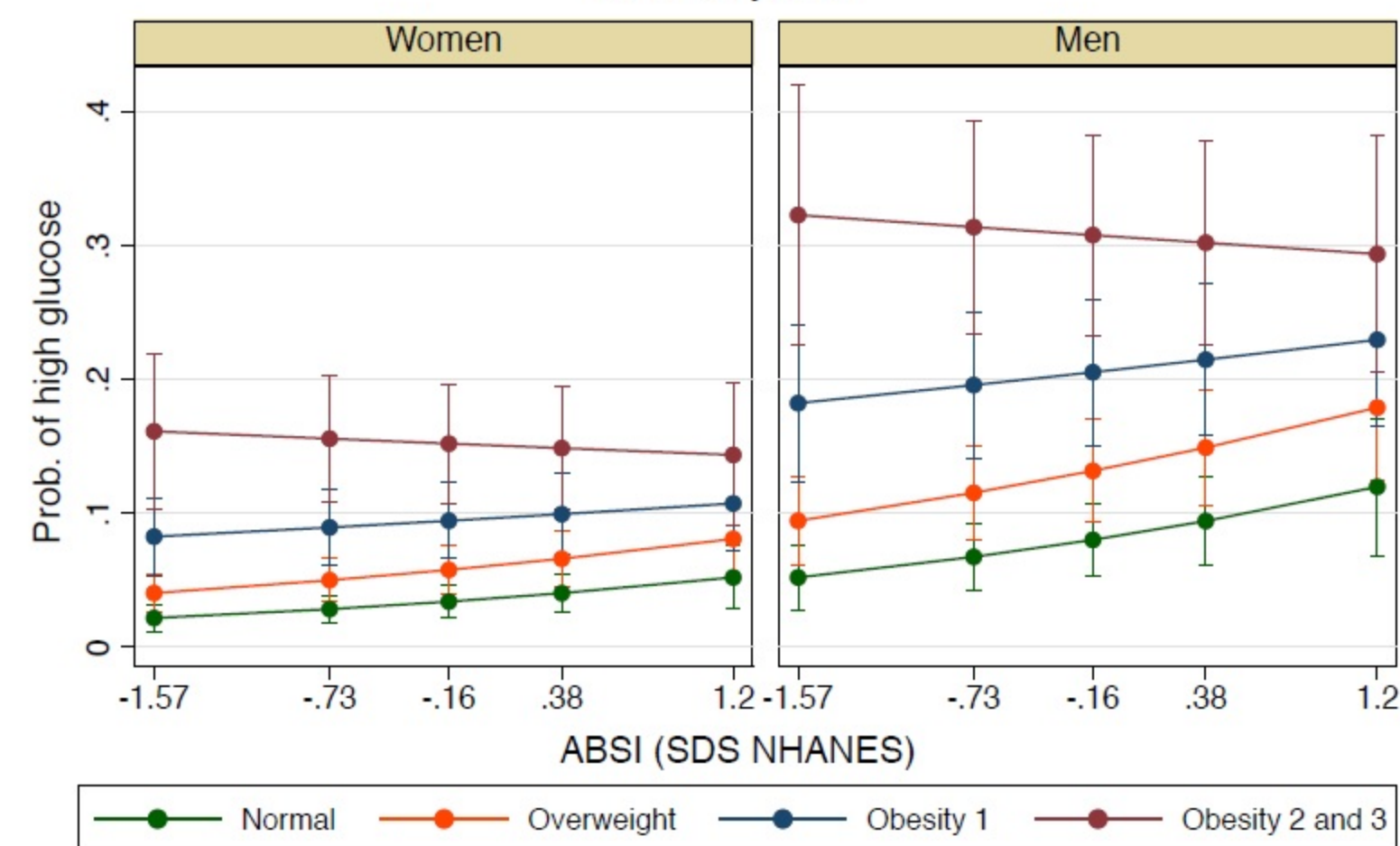

30 to 39 years

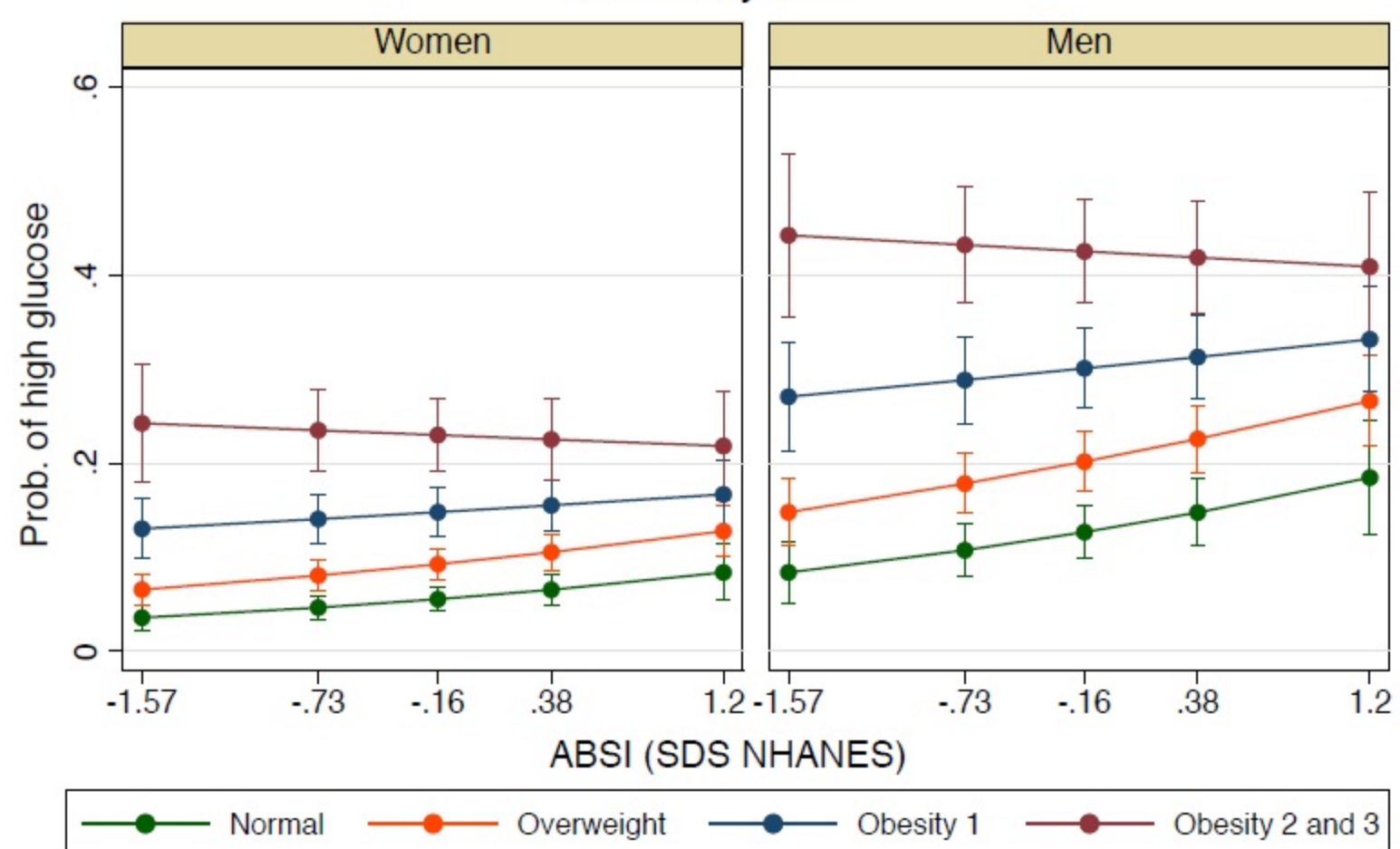

40 to 49 years

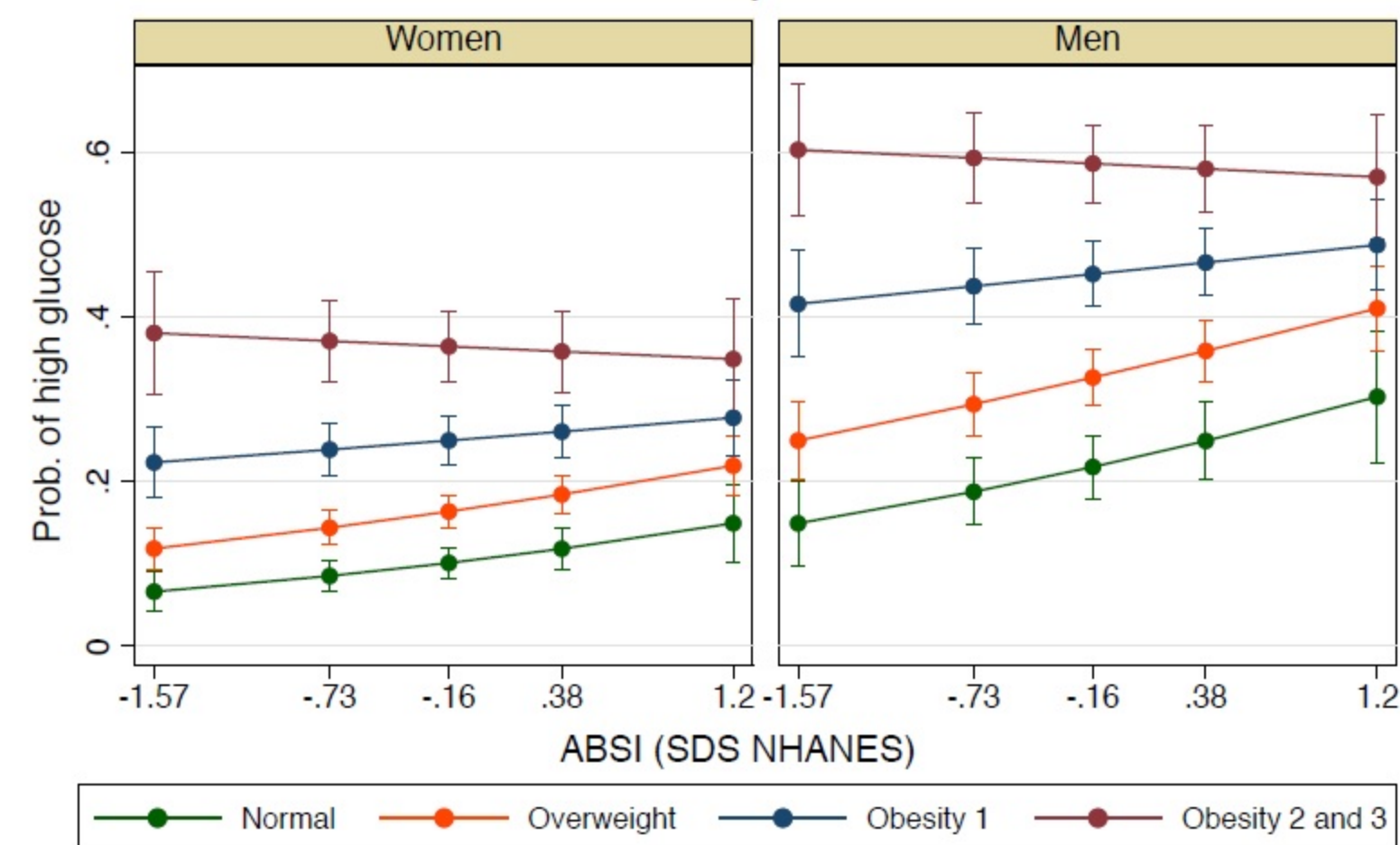

50 to 59 years

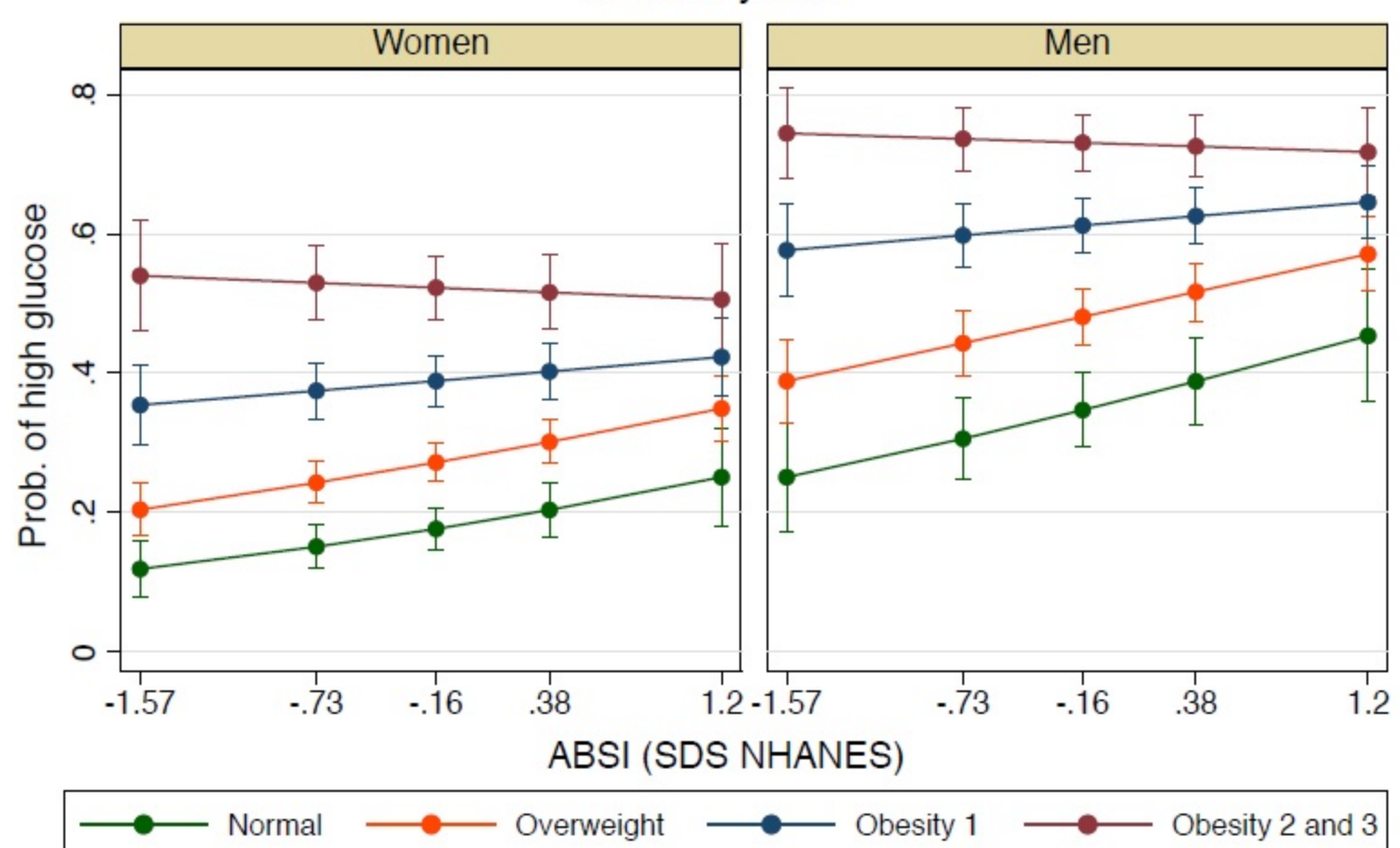

60 to 69 years

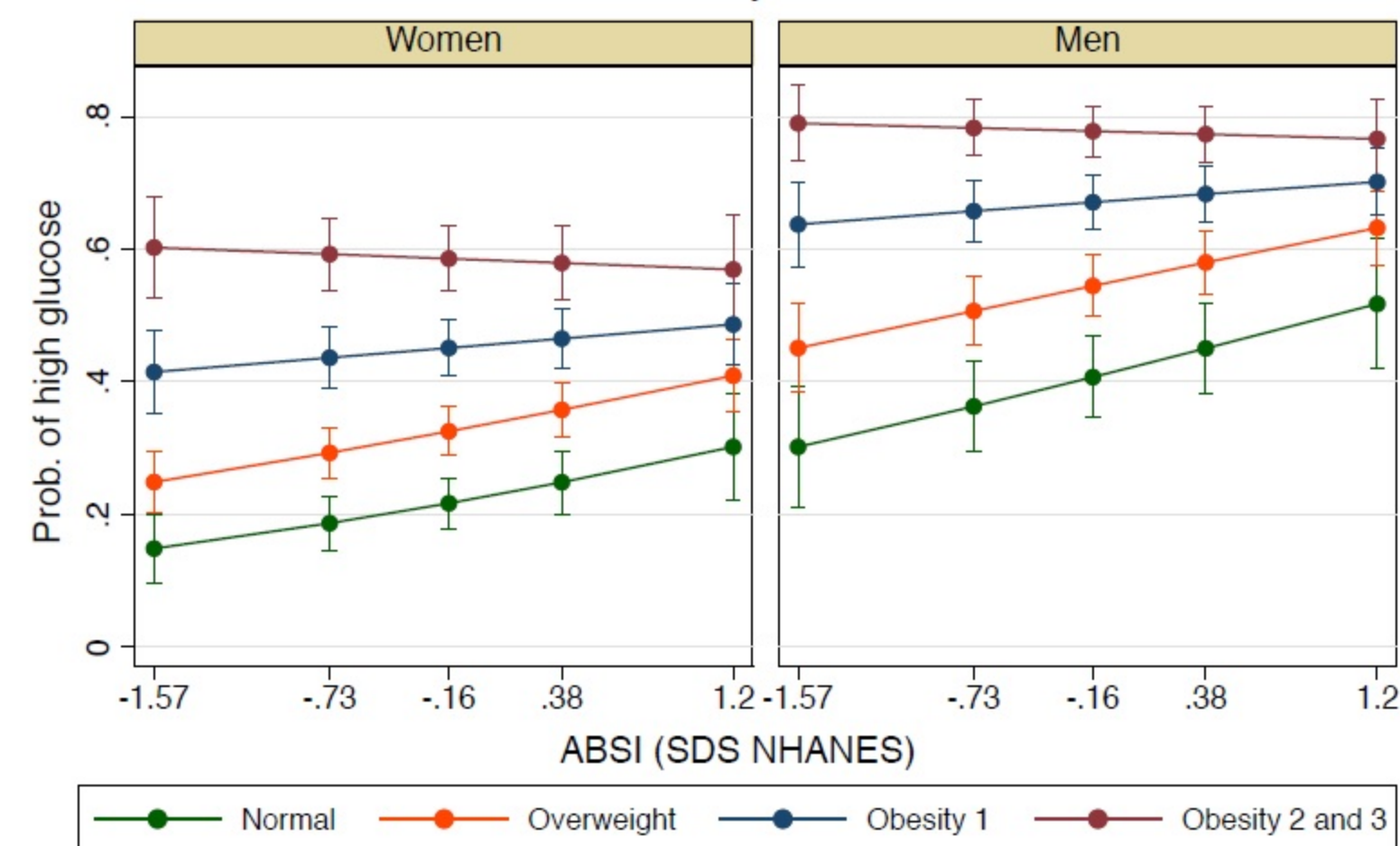

70 to 76 years

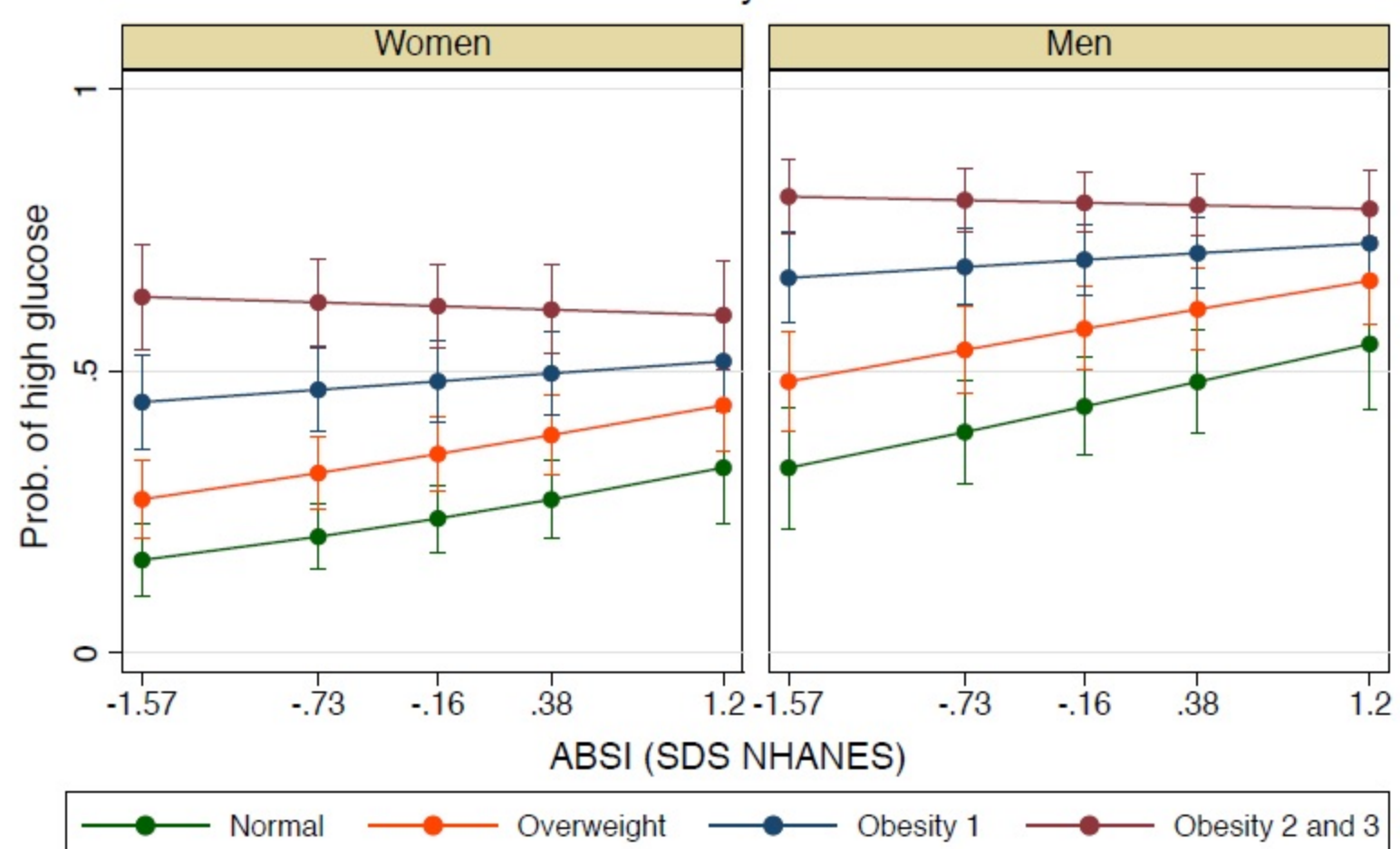

Supplement: S4 Fig — The values of ABSI correspond to the internal 5th, 25th, 50th, 75th and 95th percentiles. Values are probabilities and 95% confidence intervals. (PDF) [file pone.0185013.s004.pdf]

19 to 29 years

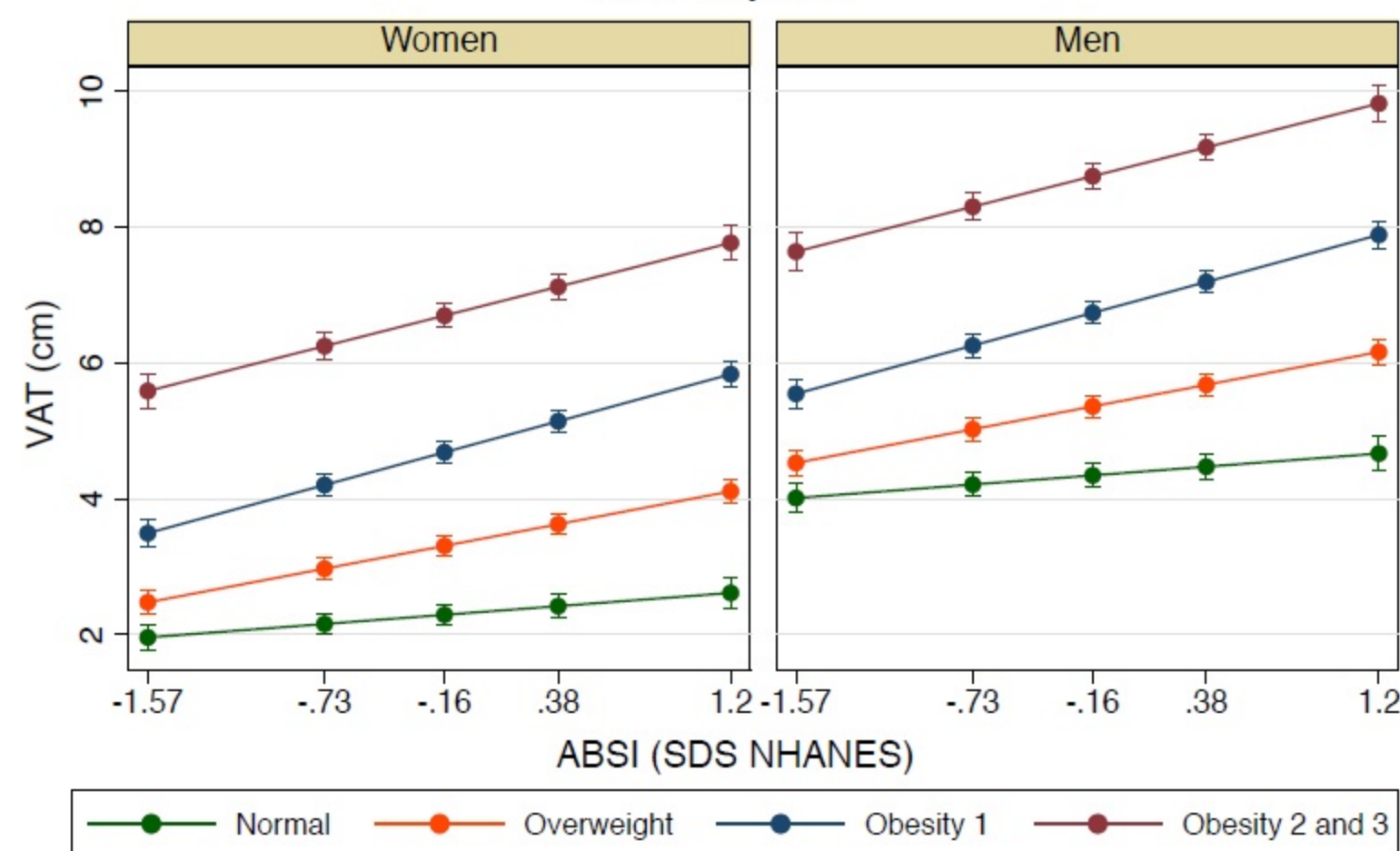

30 to 39 years

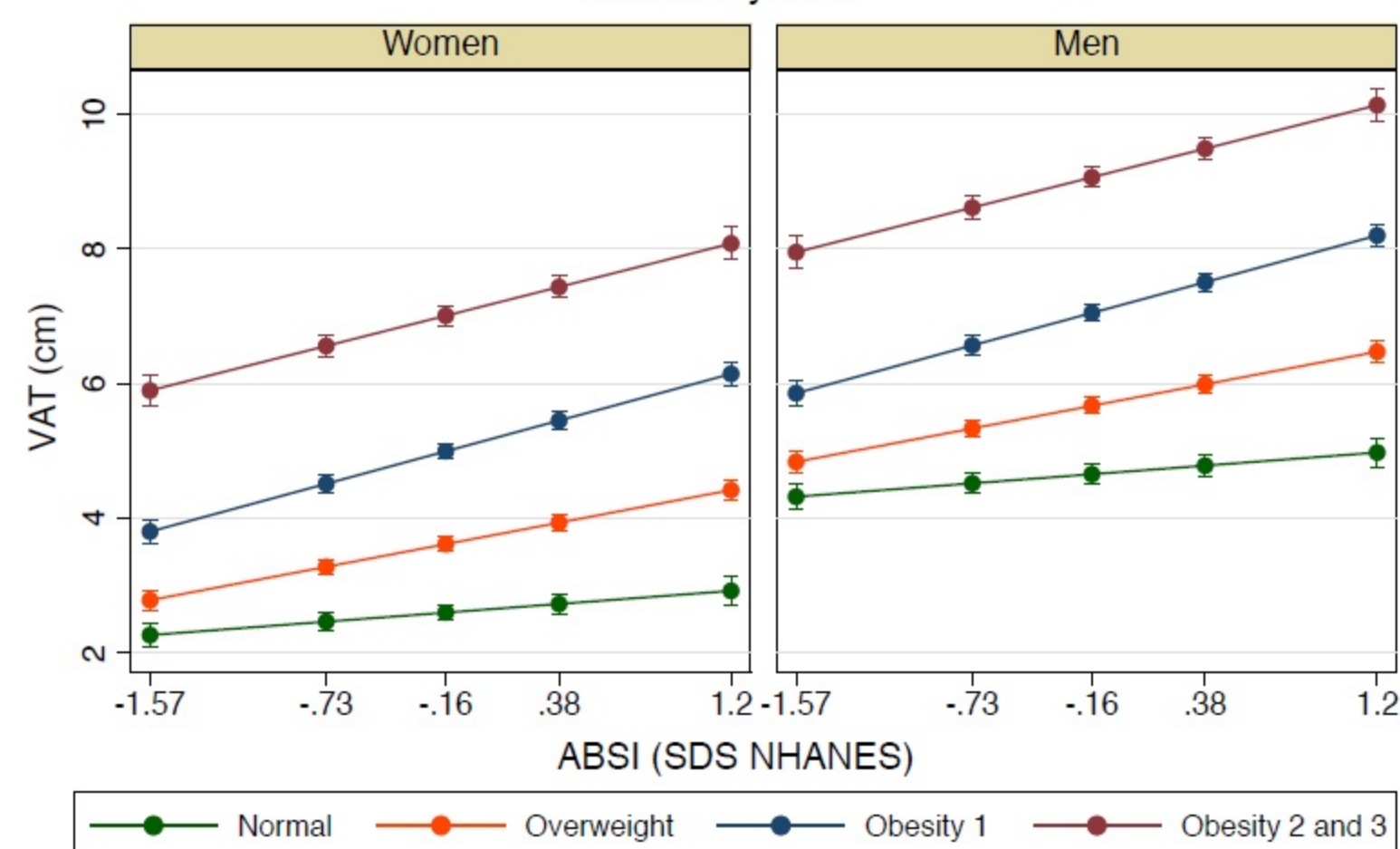

40 to 49 years

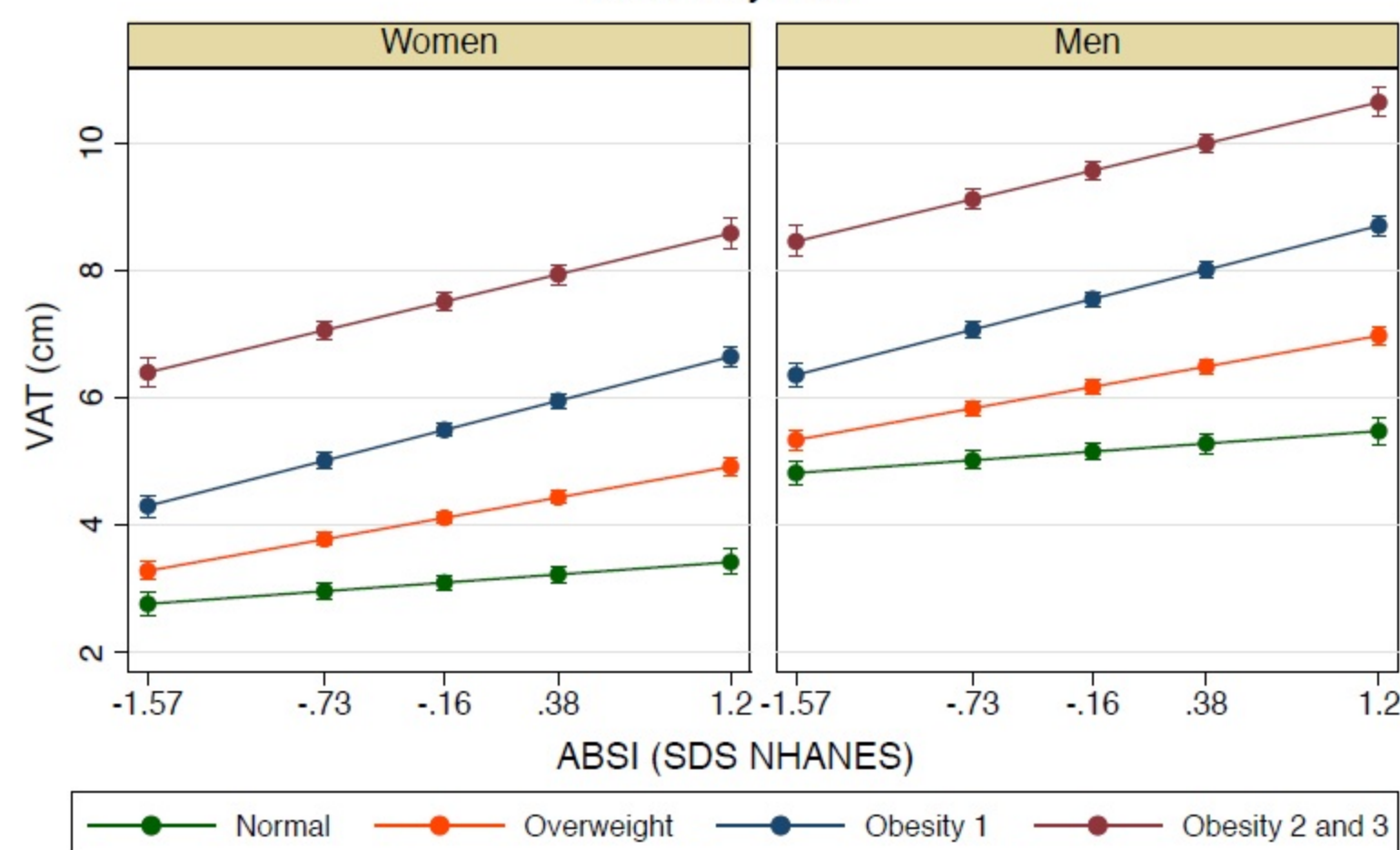

50 to 59 years

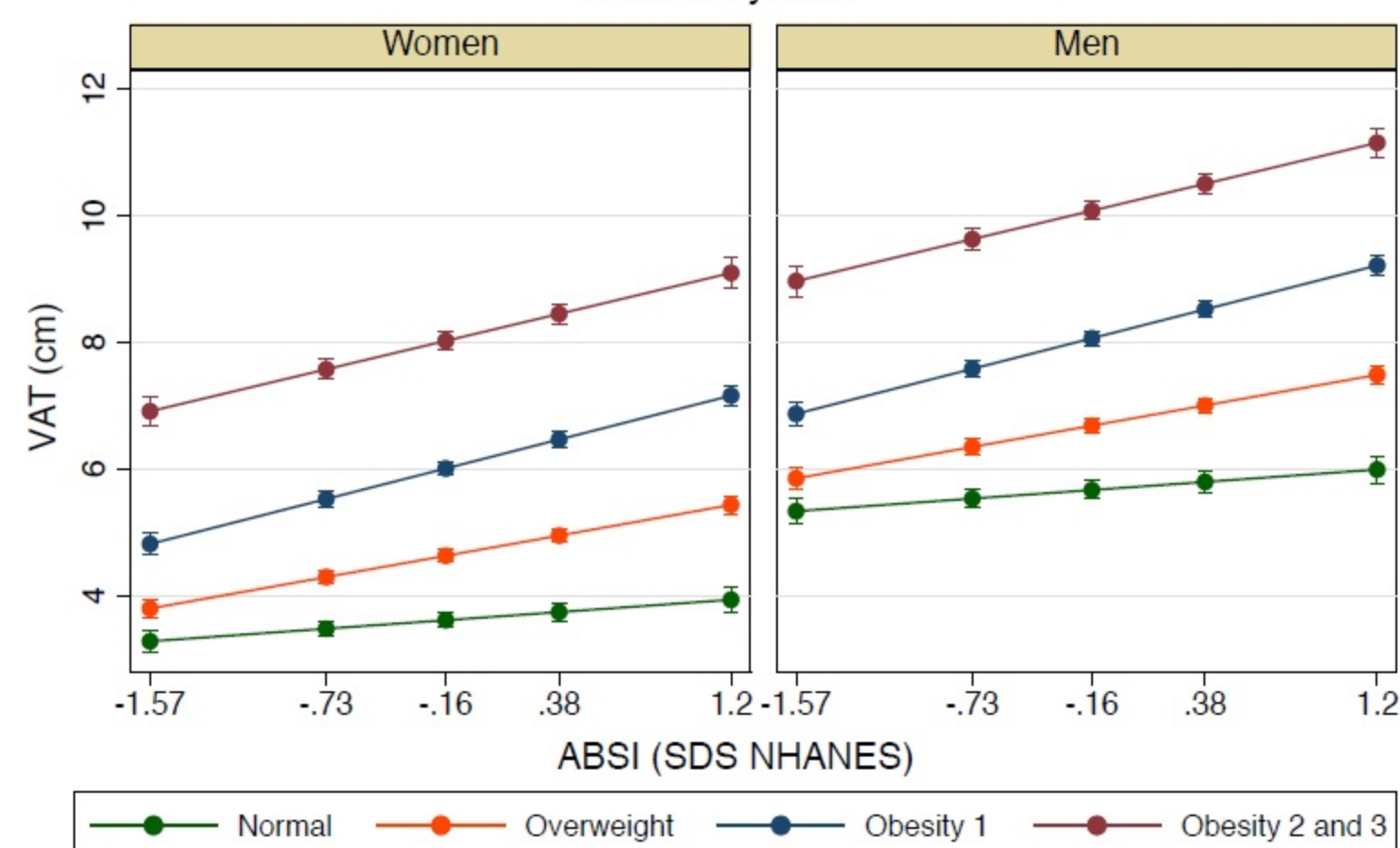

60 to 69 years

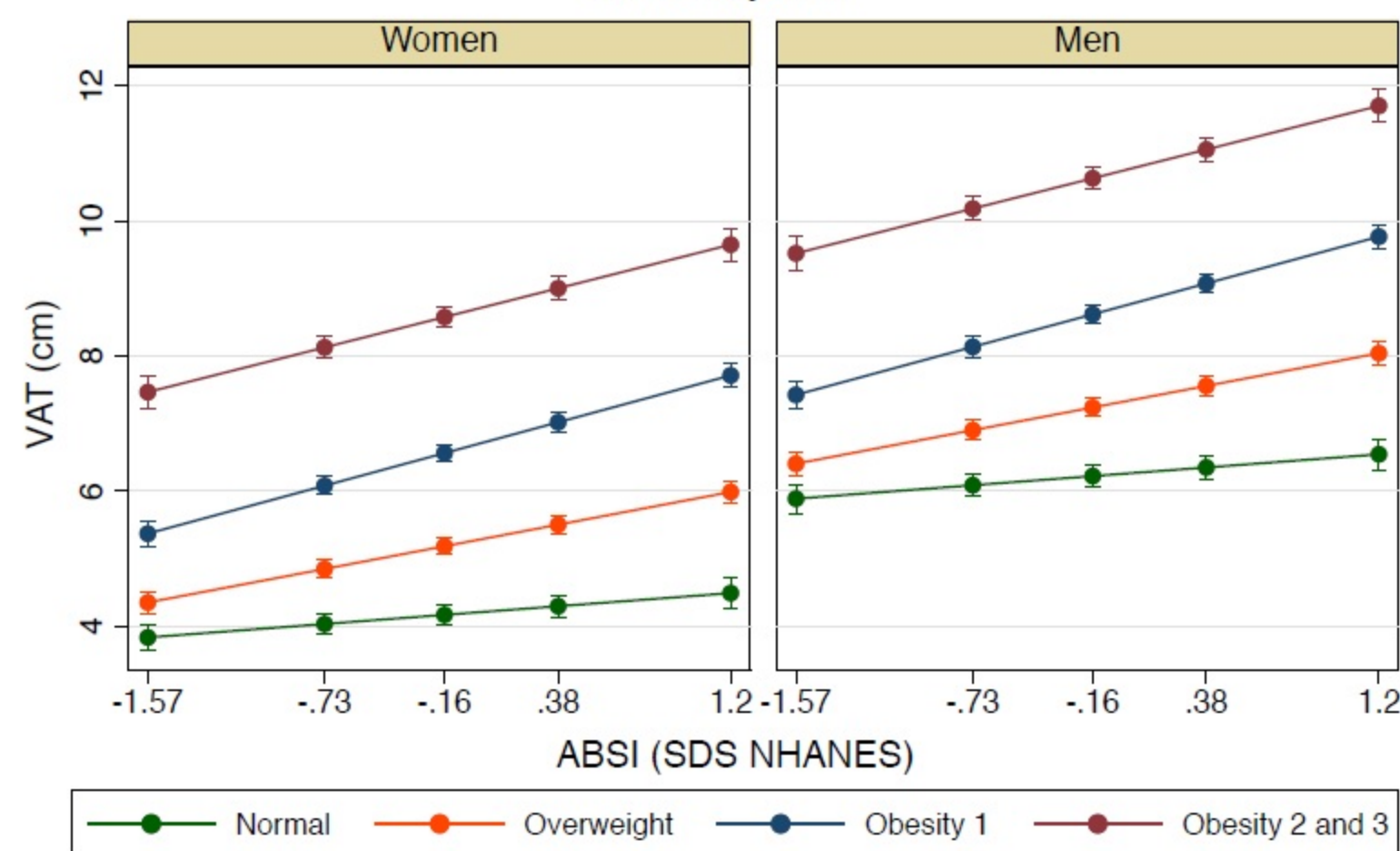

70 to 76 years

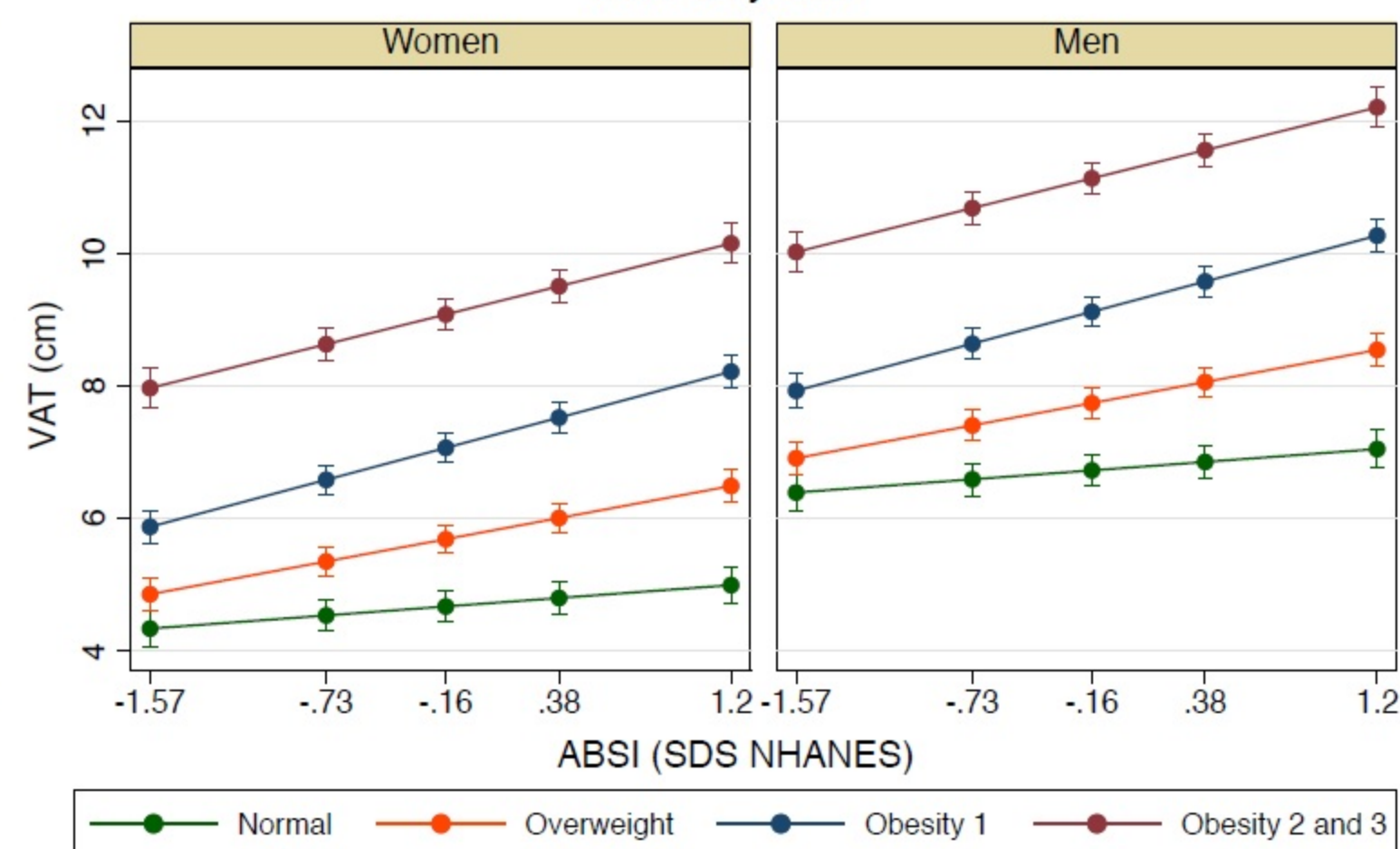

Supplement: S5 Fig — The values of ABSI correspond to the internal 5th, 25th, 50th, 75th and 95th percentiles. Values are predicted means and 95% confidence intervals. (PDF) [file pone.0185013.s005.pdf]
