# Supplementary material for: Association of Body Shape Index (ABSI) with cardio-metabolic risk factors: A cross-sectional study of 6081 Caucasian adults
Source: PLoS One. 2017 Sep 25;12(9):e0185013. doi: 10.1371/journal.pone.0185013 (PMC5612697; doi:10.1371/journal.pone.0185013)
Supplement: S1 Table — (DOCX) [file pone.0185013.s006.docx]

| **High triglycerides** | | | | |
| --- | --- | --- | --- | --- |
|  | A1 | A2 | A3 | A4 |
| **ABSI** |  |  |  |  |
| ABSI (SDS NHANES) | 0.50** | 0.31*** | 0.33*** |  |
|  | [0.17,0.82] | [0.20,0.41] | [0.23,0.44] |  |
| **BMI Classes** |  |  |  |  |
| Normal | Ref. | Ref. |  | Ref. |
| Overweight | 0.55*** | 0.57*** |  | 0.61*** |
|  | [0.26,0.84] | [0.28,0.86] |  | [0.32,0.90] |
| Obesity 1 | 1.12*** | 1.14*** |  | 1.20*** |
|  | [0.83,1.42] | [0.85,1.44] |  | [0.90,1.49] |
| Obesity 2 and 3 | 1.34*** | 1.37*** |  | 1.40*** |
|  | [1.00,1.67] | [1.04,1.70] |  | [1.07,1.73] |
| **BMI-ABSI interaction** |  |  |  |  |
| Normal # ABSI (SDS NHANES) | Ref. |  |  |  |
| Overweight # ABSI (SDS NHANES) | -0.11 |  |  |  |
|  | [-0.47,0.25] |  |  |  |
| Obesity 1 # ABSI (SDS NHANES) | -0.26 |  |  |  |
|  | [-0.63,0.10] |  |  |  |
| Obesity 2 and 3 # ABSI (SDS NHANES) | -0.35 |  |  |  |
|  | [-0.77,0.07] |  |  |  |
| **Age Classes** |  |  |  |  |
| 19 to 29 years | Ref. | Ref. | Ref. | Ref. |
| 30 to 39 years | -0.11 | -0.10 | -0.11 | -0.08 |
|  | [-0.44,0.23] | [-0.43,0.23] | [-0.44,0.21] | [-0.40,0.25] |
| 40 to 49 years | 0.02 | 0.03 | 0.03 | 0.06 |
|  | [-0.29,0.34] | [-0.28,0.35] | [-0.27,0.34] | [-0.25,0.37] |
| 50 to 59 years | 0.16 | 0.17 | 0.20 | 0.19 |
|  | [-0.17,0.49] | [-0.16,0.50] | [-0.12,0.53] | [-0.13,0.52] |
| 60 to 69 years | 0.33 | 0.36 | 0.41* | 0.37 |
|  | [-0.05,0.71] | [-0.02,0.73] | [0.04,0.78] | [-0.00,0.74] |
| 70 to 76 years | 0.45 | 0.46 | 0.56 | 0.45 |
|  | [-0.17,1.07] | [-0.16,1.08] | [-0.05,1.18] | [-0.16,1.07] |
| **Sex** |  |  |  |  |
| Women | Ref. | Ref. | Ref. | Ref. |
| Men | 1.18*** | 1.17*** | 1.32*** | 1.27*** |
|  | [1.01,1.36] | [0.99,1.34] | [1.15,1.49] | [1.10,1.44] |
|  |  |  |  |  |
| Constant | -2.91*** | -2.94*** | -2.25*** | -3.06*** |
|  | [-3.27,-2.54] | [-3.30,-2.58] | [-2.53,-1.96] | [-3.41,-2.70] |
| Observations | 4800 | 4800 | 4800 | 4800 |
| BIC | 3722 | 3701 | 3778 | 3726 |

**Table S1. Association between ABSI, BMI and metabolic syndrome components in subjects without drug therapy**

|  | **Low HDL** |  |  |  |
| --- | --- | --- | --- | --- |
|  | B1 | B2 | B3 | B4 |
| **ABSI** |  |  |  |  |
| ABSI (SDS NHANES) | 0.55*** | 0.22*** | 0.25*** |  |
|  | [0.25,0.84] | [0.12,0.31] | [0.15,0.34] |  |
| **BMI Classes** |  |  |  |  |
| Normal | Ref. | Ref. |  | Ref. |
| Overweight | 0.69*** | 0.75*** |  | 0.77*** |
|  | [0.42,0.95] | [0.48,1.01] |  | [0.51,1.04] |
| Obesity 1 | 1.32*** | 1.38*** |  | 1.42*** |
|  | [1.05,1.60] | [1.11,1.66] |  | [1.15,1.69] |
| Obesity 2 and 3 | 1.63*** | 1.71*** |  | 1.73*** |
|  | [1.32,1.94] | [1.41,2.02] |  | [1.42,2.04] |
| **BMI-ABSI interaction** |  |  |  |  |
| Normal # ABSI (SDS NHANES) | Ref. |  |  |  |
| Overweight # ABSI (SDS NHANES) | -0.34* |  |  |  |
|  | [-0.67,-0.01] |  |  |  |
| Obesity 1 # ABSI (SDS NHANES) | -0.37* |  |  |  |
|  | [-0.70,-0.03] |  |  |  |
| Obesity 2 and 3 # ABSI (SDS NHANES) | -0.47* |  |  |  |
|  | [-0.85,-0.09] |  |  |  |
| **Age classes** |  |  |  |  |
| 19 to 29 years | Ref. | Ref. | Ref. | Ref. |
| 30 to 39 years | 0.05 | 0.06 | 0.05 | 0.09 |
|  | [-0.23,0.33] | [-0.22,0.34] | [-0.22,0.33] | [-0.19,0.36] |
| 40 to 49 years | -0.25 | -0.24 | -0.21 | -0.19 |
|  | [-0.52,0.02] | [-0.51,0.04] | [-0.48,0.05] | [-0.46,0.08] |
| 50 to 59 years | -0.52*** | -0.51*** | -0.43** | -0.47** |
|  | [-0.82,-0.22] | [-0.81,-0.21] | [-0.72,-0.14] | [-0.77,-0.17] |
| 60 to 69 years | -0.65*** | -0.63** | -0.51** | -0.60** |
|  | [-1.03,-0.28] | [-1.01,-0.25] | [-0.88,-0.14] | [-0.98,-0.23] |
| 70 to 76 years | -0.48 | -0.48 | -0.31 | -0.47 |
|  | [-1.15,0.18] | [-1.14,0.19] | [-0.96,0.35] | [-1.13,0.20] |
| **Sex** |  |  |  |  |
| Women | Ref. | Ref. | Ref. | Ref. |
| Men | 0.21* | 0.19* | 0.40*** | 0.27** |
|  | [0.03,0.38] | [0.01,0.37] | [0.23,0.57] | [0.10,0.45] |
|  |  |  |  |  |
| Constant | -2.32*** | -2.39*** | -1.54*** | -2.50*** |
|  | [-2.64,-2.00] | [-2.70,-2.07] | [-1.78,-1.31] | [-2.81,-2.18] |
| Observations | 4800 | 4800 | 4800 | 4800 |
| BIC | 4086 | 4067 | 4218 | 4077 |

| **High blood pressure** | | | | |
| --- | --- | --- | --- | --- |
|  | C1 | C2 | C3 | C4 |
| **ABSI** |  |  |  |  |
| ABSI (SDS NHANES) | 0.08 | 0.08 | 0.11** |  |
|  | [-0.13,0.29] | [-0.00,0.16] | [0.03,0.19] |  |
| **BMI classes** |  |  |  |  |
| Normal | Ref. | Ref. |  | Ref. |
| Overweight | 0.68*** | 0.68*** |  | 0.69*** |
|  | [0.47,0.88] | [0.49,0.87] |  | [0.49,0.88] |
| Obesity 1 | 1.40*** | 1.39*** |  | 1.40*** |
|  | [1.18,1.61] | [1.18,1.60] |  | [1.20,1.61] |
| Obesity 2 and 3 | 2.26*** | 2.25*** |  | 2.26*** |
|  | [1.98,2.53] | [1.99,2.51] |  | [1.99,2.52] |
| **BMI-ABSI interaction** |  |  |  |  |
| Normal # ABSI (SDS NHANES) | Ref. |  |  |  |
| Overweight # ABSI (SDS NHANES) | -0.03 |  |  |  |
|  | [-0.28,0.21] |  |  |  |
| Obesity 1 # ABSI (SDS NHANES) | 0.06 |  |  |  |
|  | [-0.20,0.32] |  |  |  |
| Obesity 2 and 3 # ABSI (SDS NHANES) | 0.02 |  |  |  |
|  | [-0.30,0.35] |  |  |  |
| **Age classes** |  |  |  |  |
| 19 to 29 years | Ref. | Ref. | Ref. | Ref. |
| 30 to 39 years | 0.30* | 0.30* | 0.27* | 0.31* |
|  | [0.03,0.58] | [0.02,0.58] | [0.01,0.53] | [0.03,0.58] |
| 40 to 49 years | 0.84*** | 0.83*** | 0.78*** | 0.85*** |
|  | [0.57,1.10] | [0.57,1.10] | [0.53,1.03] | [0.59,1.11] |
| 50 to 59 years | 1.25*** | 1.24*** | 1.21*** | 1.26*** |
|  | [0.98,1.52] | [0.97,1.52] | [0.95,1.47] | [0.99,1.53] |
| 60 to 69 years | 1.86*** | 1.85*** | 1.83*** | 1.86*** |
|  | [1.54,2.18] | [1.54,2.17] | [1.53,2.13] | [1.55,2.18] |
| 70 to 76 years | 2.48*** | 2.47*** | 2.48*** | 2.46*** |
|  | [1.91,3.04] | [1.90,3.04] | [1.93,3.03] | [1.90,3.03] |
| **Sex** |  |  |  |  |
| Women | Ref. | Ref. | Ref. | Ref. |
| Men | 1.29*** | 1.29*** | 1.44*** | 1.32*** |
|  | [1.13,1.44] | [1.14,1.45] | [1.29,1.58] | [1.17,1.47] |
|  |  |  |  |  |
| Constant | -2.62*** | -2.61*** | -1.70*** | -2.65*** |
|  | [-2.91,-2.33] | [-2.90,-2.33] | [-1.93,-1.47] | [-2.93,-2.37] |
| Observations | 4800 | 4800 | 4800 | 4800 |
| BIC | 5355 | 5330 | 5702 | 5325 |

| **High fasting glucose** | | | | |
| --- | --- | --- | --- | --- |
|  | D1 | D2 | D3 | D4 |
| **ABSI** |  |  |  |  |
| ABSI (SDS NHANES) | 0.33* | 0.19*** | 0.21*** |  |
|  | [0.07,0.58] | [0.10,0.28] | [0.12,0.30] |  |
| **BMI classes** |  |  |  |  |
| Normal | Ref. | Ref. |  | Ref. |
| Overweight | 0.58*** | 0.60*** |  | 0.62*** |
|  | [0.35,0.81] | [0.37,0.83] |  | [0.40,0.85] |
| Obesity 1 | 1.05*** | 1.08*** |  | 1.11*** |
|  | [0.81,1.29] | [0.85,1.32] |  | [0.87,1.35] |
| Obesity 2 and 3 | 1.46*** | 1.52*** |  | 1.53*** |
|  | [1.18,1.74] | [1.25,1.80] |  | [1.26,1.81] |
| **BMI-ABSI interaction** |  |  |  |  |
| Normal # ABSI (SDS NHANES) | Ref. |  |  |  |
| Overweight # ABSI (SDS NHANES) | -0.04 |  |  |  |
|  | [-0.33,0.25] |  |  |  |
| Obesity 1 # ABSI (SDS NHANES) | -0.22 |  |  |  |
|  | [-0.51,0.08] |  |  |  |
| Obesity 2 and 3 # ABSI (SDS NHANES) | -0.36* |  |  |  |
|  | [-0.70,-0.01] |  |  |  |
| **Age classes** |  |  |  |  |
| 19 to 29 years | Ref. | Ref. | Ref. | Ref. |
| 30 to 39 years | 0.58** | 0.58** | 0.56** | 0.60** |
|  | [0.21,0.94] | [0.21,0.95] | [0.20,0.93] | [0.23,0.96] |
| 40 to 49 years | 1.18*** | 1.19*** | 1.17*** | 1.21*** |
|  | [0.83,1.53] | [0.84,1.54] | [0.82,1.51] | [0.86,1.56] |
| 50 to 59 years | 1.76*** | 1.77*** | 1.76*** | 1.78*** |
|  | [1.40,2.11] | [1.41,2.12] | [1.41,2.11] | [1.43,2.14] |
| 60 to 69 years | 1.89*** | 1.91*** | 1.94*** | 1.92*** |
|  | [1.50,2.28] | [1.53,2.30] | [1.56,2.32] | [1.53,2.30] |
| 70 to 76 years | 1.83*** | 1.84*** | 1.91*** | 1.83*** |
|  | [1.26,2.40] | [1.27,2.41] | [1.35,2.48] | [1.26,2.40] |
| **Sex** |  |  |  |  |
| Women | Ref. | Ref. | Ref. | Ref. |
| Men | 0.87*** | 0.85*** | 1.01*** | 0.91*** |
|  | [0.71,1.03] | [0.70,1.01] | [0.86,1.16] | [0.76,1.07] |
|  |  |  |  |  |
| Constant | -3.34*** | -3.38*** | -2.66*** | -3.45*** |
|  | [-3.72,-2.96] | [-3.75,-3.00] | [-2.99,-2.33] | [-3.82,-3.07] |
| Observations | 4800 | 4800 | 4800 | 4800 |
| BIC | 4699 | 4681 | 4809 | 4690 |

Values are regression coefficients, 95% confidence intervals in brackets and BIC.

Model 1: ABSI (continuous), BMI (discrete), ABSI·BMI interaction (continuous X discrete), sex (discrete), age (discrete).

Model 2: same as #1 without the ABSI·BMI interaction.

Model 3: same as #2 without BMI.

Model 4: same as #2 without ABSI.
Abbreviations: Ref = Reference category (regression coefficient = 0)

* p<0.05, ** p<0.01, *** p<0.001
